# Analysis of Twitter data with the Bayesian fused graphical lasso

**DOI:** 10.1371/journal.pone.0235596

**Published:** 2020-07-27

**Authors:** Mehran Aflakparast, Mathisca de Gunst, Wessel van Wieringen

**Affiliations:** 1 Department of Mathematics, Vrije Universiteit Amsterdam, Amsterdam, The Netherlands; 2 Department of Epidemiology and Data Science, Amsterdam Public Health Research Institute, Amsterdam UMC, location VUmc, Amsterdam, The Netherlands; Yunnan University of Finance and Economics, CHINA

## Abstract

We propose a method to simplify textual Twitter data into understandable networks of terms that can signify important events and their possible changes over time. The method allows for common characteristics of the networks across time periods and each period can comprise multiple unknown sub-networks. The networks are described by Gaussian graphical models and their parameter values are estimated through a Bayesian approach with a *fused lasso*-type prior on the precision matrices of the underlying mixtures of the sub-models. A flexible data allocation scheme is at the heart of an MCMC algorithm to recover mean and covariance parameters of the mixture components. Several implementations of the outlined estimation procedure are studied and compared based on simulated data. The procedure with the highest predictive power is used for mining tweets regarding the 2009 Iranian presidential election.

## 1 Introduction

Twitter is a prominent social media tool that provides a rich resource of information. The huge volume of gathered information calls for powerful methods to translate large and complex data into small chunks of understandable signals that can be used in several areas ranging from social sciences and health research to marketing and e-commerce. As an example, studied later in detail, more than one million tweets related to the social upheaval surrounding the 2009 Iranian presidential election may be compressed into an accessible visual summary. Such summary information can entail different topics that are highlighted in a certain period of time and have evolved over time. This can be viewed as a form of network reconstruction where collections of linked words, concepts or terms represent highlighted topics at a certain time-stamp. Any changes in such topics over time, from becoming outdated, expanded or created, can be explained by evolution of the links between the words.

Our interest is in the reconstruction of networks of words/terms from *multiple* Twitter data sets corresponding to specific time periods, where different networks share topological similarities. Next to this, data from a particular time period may be heterogeneous in that they cannot be represented by a single network. This phenomenon might be explained by differences among (parts of) the networks across different time periods, or can originate from hidden sub-networks within each time period. Naturally, to end up with interpretable networks we aim to reconstruct networks in which only a few terms or predictors play an important role. This means that we search for *sparse* networks, networks with relatively few links. Therefore we *a)* propose a framework to simultaneously reconstruct multiple sparse networks with a possibly shared structure and *b)* extend this idea to the case where there is more than one network for a given time period.

The problem of network reconstruction is operationalized here as the estimation of a number of Gaussian graphical models (GGMs) for which the nonzero elements of the precision matrices (inverse covariance matrices) correspond to edges of the network. Estimation of a single GGM, especially in a high-dimensional setting where the data dimension is larger than the sample size, often proceeds in a regularized fashion (see, for example, [1–5]). These methods typically minimize the log-likelihood of the data augmented with an *ℓ*_1_-penalty on the elements of the precision matrix. For instance, Meinshausen and Bühlmann [1] proposed to identify the edges of a GGM by sparse estimation of the precision matrix through lasso regressions of each random variable on all other variables. Friedman et al. [4] presented the *graphical lasso*, a procedure to estimate sparsely the precision matrix directly. In [2, 3, 5] different estimation algorithms for the graphical lasso method are treated. An alternative approach is provided in [6], where it is illustrated that in cases where the true graphical model does not need to be extremely sparse in terms of containing many zero elements, ridge penalties coupled with post-hoc selection may outperform the lasso. A common challenge with these approaches is that they do not take into consideration the uncertainty of the parameter estimates and require selection of the penalty parameters that control sparsity. Wang [7] proposed a Bayesian counterpart to the graphical lasso that provides a solution for such shortcomings, and developed a fast algorithm to estimate a moderately large precision matrix. However, this method does not serve our purpose in the face of multiple networks with possibly evolving structures.

There is a number of methods that consider simultaneous estimation of multiple graphical models corresponding to more than one data set (see for instance [8–12]). Guo et al. [8] extended the graphical lasso with a certain parametrization of multiple precision matrices whose elements are expressed as a product of shared and class-specific factors. Through a hierarchical penalty on both the shared and the class-specific factors their method shrinks some elements in the inverse covariance matrices to zero. Danaher et al. [9] proposed a general framework with arbitrary type of penalty and derived fused lasso and group lasso estimators, where the fused lasso estimation encourages shared structure and/or equal values for the elements across the precision matrices, while the group lasso estimation emphasizes only a shared sparse structure. More recently, a fused ridge version of multiple graphical model estimation has been proposed [11]. As another example, Zhu et al. [10] adapted the truncated *ℓ*_1_-penalization of [13] to stimulate elements of the precision matrices across data sets to be similar. Bayesian counterparts include [12, 14]. These methods give proper consideration to common characteristics of the data sets while simultaneously estimating them. However, they lack the flexibility to account for heterogeneity within each data set. In [15] the problem of learning the evolution of an interaction network, modeled as a GGM, from cross-sectional, high-dimensional data in the face of heterogeneity was addressed through fused ridge penalized estimation of a combination of mixtures of GGMs. Here we consider this problem from a Bayesian perspective.

In this paper we present a novel Bayesian approach to the joint estimation of multiple graphical models, that takes into account both shared topological structures between the networks, and heterogeneity within the networks. In particular, we propose a Bayesian Gaussian fused graphical lasso estimation algorithm to estimate group-wise precision matrices that may exhibit network similarities, and augment this with a mixture model to account for heterogeneity of the data within a network. This is done in the spirit of the data integrative Bayesian inference method that we proposed in [16], by forming a new prior distribution on the elements of the precision matrices and obtaining a posterior distribution that resembles the *ℓ*_1_-penalized likelihood plus a fused penalty. A data allocation scheme is employed to simultaneously uncover the hidden clustering components of the mixture model while estimation of the cluster-specific precision matrices is achieved through column-wise block Gibbs sampling. In the application that we consider the different networks originate from multiple time periods, however, for the method the ordering over time is irrelevant. This makes our method widely applicable.

The paper is organized as follows. In Section 2.1 we propose a Bayesian Gaussian graphical network reconstruction method for data from multiple time periods or multiple groups. This is extended in Section 2.2 to allow for heterogeneity in the sense that each time period may encompass data from more than one (unknown) sub-population. In Section 3.1, these approaches are evaluated and compared by simulation. Section 3.2 illustrates the application of the proposed method in analyzing tweets regarding the 2009 Iranian presidential election. We conclude in Section 4 with discussing future improvements.

## 2 Materials and methods

Throughout the paper we will use capital letters to denote random variables, random vectors or random matrices; bold type will be used for vectors and matrices. The symbol ∝ stands for “is proportional to”. To emphasize that for the proposed method the ordering of the time periods is irrelevant, throughout this section we will use the word *group* instead of time period, and the unknown sub-populations belonging to one time period, will be called *subgroups*, *clusters*, or *components*.

### 2.1 Bayesian fused graphical lasso

Characteristics from a sample of *n* individuals comprising *T* groups have been observed. For *t* = 1, …, *T*, the number of individuals in group *t* will be denoted by *n*_*t*_, and for $i=1,\ldots ,n_{1},n_{1}+1,\ldots ,n_{1}+n_{2},n_{1}+n_{2}+1,\ldots ,\sum_{t=1}^{T}n_{t}=n$, the random vector **Y**_*i*_ represents the *p*-dimensional vector of characteristics of individual *i*. In the sequel we will write $Y=(Y_{1}^{T},\ldots ,Y_{n}^{T})$ for the complete *n* × *p* data matrix. For later use we also define $n_{<t}=\sum_{s=1}^{t-1}n_{s}$ and *n*_<1_ = 0. The grouping of individuals is exhaustive and exclusive in the sense that an individual appears in a single group only.

The random vectors of characteristics are assumed to be independent and to follow a group-wise Gaussian law,

$$\begin{array}{c} Y_{i}∼N_{p}\left(\mu_{t},\Omega_{t}^{-1}\right),\;i=n_{<t}+1,\ldots ,n_{<t}+n_{t},\;t=1,\ldots ,T. \end{array}$$

We consider the joint estimation the groups’ precision matrices **Ω** = {**Ω**_1_, ‥, **Ω**_*T*_}. For the purpose of interpretability sparse estimates are sought for, while the context suggests that the structure of the precision matrices may be shared between groups. In a frequentist setting all requirements (high-dimensionality, sparsity and a possibly common structure of the precision matrices) are catered for by the fused graphical lasso estimator [9], which maximizes the following penalized joint log-likelihood

$$\begin{array}{c} \sum_{t=1}^{T}\text{log}(|\Omega_{t}\left|\right)-\text{tr}\left(S_{t}\Omega_{t}\right)-\sum_{t=1}^{T}\lambda_{t}∥\Omega_{t}{∥}_{1}-\sum_{t_{1}<t_{2}}\lambda_{t_{1},t_{2}}{∥\Omega_{t_{1}}-\Omega_{t_{2}}∥}_{1}, \end{array}$$ (1)

with respect to **Ω**. In (1), **S**_*t*_ = **S**_*t*_(**Y**) denotes the sample covariance matrix of group *t*. Note that the estimator above generalizes the one originally proposed in [9] which uses $\lambda_{t_{1},t_{2}}=\lambda_{f}$ for all *t*_1_ and *t*_2_ (except *t*_1_ ≠ *t*_2_). The last two summands of the penalized log-likelihood (1) comprise the fused graphical lasso penalty. The convexity of the penalty tackles the high-dimensionality, while its first summand (i.e., the lasso penalty) induces sparsity, and, finally, its second summand (i.e., the fused penalty) shrinks the precision matrices towards a common structure for large values of the penalty parameter $\lambda_{t_{1},t_{2}}$.

Here we present a Bayesian interpretation of the fused graphical lasso. This requires the evaluation of the joint posterior distribution *p*(**Ω**|**Y**, **Λ**) of the **Ω**_**t**_. To this end we denote by {**Ω**}_−*t*_ the set of precision matrices with **Ω**_*t*_ excluded, and define for *t* = 2, …, *T*, the prior distribution of each precision matrix given the others as

$$\begin{array}{c} p\left(\Omega_{t}\;|\;{\left\{\Omega \right\}}_{-t},\;\Lambda \right)\;\;\propto \prod_{j_{1}<j_{2}}\frac{\lambda_{t}}{2}\;\text{exp}(-\lambda_{t}\left|\omega_{j_{1}j_{2}}^{t}\right|)\;\prod_{j=1}^{p}\frac{\lambda_{t}}{2}\;\text{exp}(-\frac{\lambda_{t}}{2}\;\omega_{jj}^{t})\\ \times \prod_{t^{\prime }\neq t}\prod_{j_{1}<j_{2}}\frac{\lambda_{t^{\prime },t}}{2}\text{exp}(-\lambda_{t^{\prime },t}\;\left|\omega_{j_{1},j_{2}}^{t^{\prime }}-\omega_{j_{1},j_{2}}^{t}\right|)\;I\left(\Omega_{t}≻0\right). \end{array}$$ (2)

In the above $\omega_{ij}^{t}$ denotes the *i*, *j*-th element of **Ω**_*t*_. Note that the (2) is invariant to the order of conditioning. Furthermore, the diagonal and off-diagonal elements of the precision matrices are thus assumed to follow *a priori* an exponential and a double exponential distribution, respectively, (see for example [17] and [7] for similar approaches). The differences between corresponding precision elements of any pair of groups also obey a double exponential law. The term *I*(**Ω**_*t*_ ≻ 0) limits the support of the prior to the positive definite matrices. With the fused graphical lasso prior (2), the posterior distribution **Ω**_**t**_ is not a well-known standard distribution, but an efficient Gibbs sampling scheme can be designed. This extends the work of [7] for the Bayesian graphical lasso. In a nutshell, the Gibbs sampler amounts to iteratively sampling one column of **Ω**_*t*_ at the time which guarantees positive definiteness.

As a first step towards our Gibbs sampler we derive a tractable formulation of the conditional posterior of each precision matrix given the others. Application of the definition of conditional probability and subsequent insertion of the equality *p*(**Ω**|**Λ**) = *p*(**Ω**_*t*_|{**Ω**}_−*t*_, **Λ**)*p*({**Ω**}_−*t*_|**Λ**) yields

$$\begin{array}{ccc} p(\Omega_{t}|\;Y_{t},{\left\{\Omega \right\}}_{-t},\Lambda ) & \propto & p\left(Y_{t}\;|\;\Omega_{t}\right)\;p\left(\Omega_{t}\;|\;{\left\{\Omega \right\}}_{-t},\Lambda \right). \end{array}$$

An analytic expression is now readily available from the normality assumption of the data together with the prior (2). Gibbs sampling, however, is still hampered by the double exponential distributions employed in the prior of the precision elements. This is circumvented by a hierarchical representation of these distributions by a scale mixture of normal distributions [18],

$$\begin{array}{ccc} \frac{\lambda }{2}\;\text{exp}\left(-\lambda \;|\omega |\right) & = & \int_{0}^{\infty }\frac{1}{\sqrt{2\pi \tau }}\;\text{exp}(-\frac{\omega^{2}}{2\tau })\;\frac{\lambda^{2}}{2}\;\text{exp}(-\frac{\lambda^{2}\tau }{2})\;\;d\tau . \end{array}$$ (3)

Define, corresponding to each double exponential distribution in the prior, a latent scale parameter $\tau_{j_{1},j_{2}}^{t}$ in accordance with the scale mixture representation (3) above. Furthermore, let $\tau_{t}={\left\{\tau_{j_{1}j_{2}}^{t}\right\}}_{j_{1}<j_{2}}$ denote the independent latent scale parameters corresponding to group *t*, and let ***τ*** = {***τ***_1_, …, ***τ***_*T*_}.

Finally, we endow λ_*t*_ and λ_*t*′, *t*_ with independent gamma priors with shape parameter *s* and rate parameter *r*. Conditioning on the latent scale mixture parameters, and rewriting the hierarchical model, we obtain for *i* = *n*_<*t*_ + 1, …, *n*_<*t*_ + *n*_*t*_, *t* = 1, …, *T*,

$$\begin{array}{c} \left\{ \begin{array}{ccc} Y_{i}\;|\;\mu_{t},\;\Omega_{t} & ∼ & N_{p}\left(\mu_{t},\Omega_{t}^{-1}\right)\\ p(\Omega_{t}|\;{\left\{\Omega \right\}}_{-t},\Lambda ,\;\tau ) & = & \prod_{j=1}^{p}\frac{1}{2}\lambda_{t}\text{exp}\left(-\frac{1}{2}\lambda_{t}\omega_{jj}^{t}\right)\;\prod_{j_{1}<j_{2}}\phi_{0,\tau_{j_{1}j_{2}}^{g}}\left(\omega_{j_{1}j_{2}}^{t}\right)\\ & & \prod_{t^{\prime }\neq t}\prod_{j_{1}<j_{2}}\phi_{\omega_{j_{1}j_{2}}^{t^{\prime }},\tau_{j_{1}j_{2}}^{t^{\prime }}}\left(\omega_{j_{1}j_{2}}^{t}\right)\;I\left(\Omega_{t}≻0\right)\;\;\\ p(\tau_{t}|\;\Lambda )\;\; & = & \;\prod_{j_{1}<j_{2}}\frac{1}{2}\lambda_{t}^{2}\text{exp}\left(-\frac{1}{2}\lambda_{t}^{2}\tau_{j_{1}j_{2}}^{t}\right)\;\;\\ p(\tau_{t^{\prime }}|\;\Lambda )\;\; & = & \prod_{j_{1}<j_{2}}\frac{1}{2}\lambda_{t^{\prime },t}^{2}\text{exp}\left(-\frac{1}{2}\lambda_{t^{\prime },t}^{2}\tau_{j_{1}j_{2}}^{t^{\prime }}\right),\;\;t^{\prime }\neq t,\\ \lambda_{t} & ∼ & G(r,s),\\ \lambda_{t^{\prime },t} & ∼ & G\left(r,s\right)\;\text{for}\;t^{\prime }\neq t, \end{array}\right. \end{array}$$ (4)

where *ϕ*_*a*,*b*_ stands for the density function of the normal distribution with mean *a* = 1 and variance *b* = 1.

The mean parameters ***μ***_*t*_ are assumed to have independent priors (see Section 2.2.2). Integrating out the scale mixture parameters in the hierarchical model above will yield the double exponential distributions.

To arrive at an efficient posterior sampling procedure, the precision matrix posterior needs to be broken down further. For this we let $Y^{t}={\left\{Y_{i}\right\}}_{n_{<t}+1,\ldots ,n_{<t}+n_{t}}$ denote the set of all *Y*_*i*_ belonging to group *t*, *t* = 1, …, *T*. After putting all components of the hierarchical model (4) together, we find that the posterior of the precision matrix of the *t*-th group satisfies,

$$\begin{array}{c} p\left(\Omega_{t}|\;Y^{t},{\left\{\Omega \right\}}_{-t},\Lambda ,\tau \right)\;\;\propto {\left|\Omega_{t}\right|}^{n_{t}/2}\text{exp}[-\frac{1}{2}\text{tr}\left(S_{t}\Omega_{t}\right)]\;\prod_{j=1}^{p}\frac{1}{2}\lambda_{t}\text{exp}\left(-\frac{1}{2}\lambda_{t}\omega_{jj}^{t}\right)\;I\left(\Omega_{t}≻0\right)\\ \times \;\text{exp}\{-\frac{1}{2}\sum_{j_{1}<j_{2}}[{\left(\omega_{j_{1}j_{2}}^{t}\right)}^{2}{\left(A_{t}\right)}_{j_{1}j_{2}}-\;2\;\sum_{j_{1}<j_{2}}\;\omega_{j_{1}j_{2}}^{t}{\left(B_{t}\right)}_{j_{1}j_{2}}]\}, \end{array}$$ (5)

in which **A**_*t*_ and **B**_*t*_ are zero-diagonal and symmetric matrices with off-diagonal entries

$$\begin{array}{c} {\left(A_{t}\right)}_{j_{1}j_{2}}=\frac{1}{\tau_{j_{1}j_{2}}^{t}}+\sum_{t^{\prime }\neq t}\frac{1}{\tau_{j_{1}j_{2}}^{t^{\prime }}}\;\text{and}\;{\left(B_{t}\right)}_{j_{1}j_{2}}=\sum_{t^{\prime }\neq t}\frac{\omega_{j_{1}j_{2}}^{t^{\prime }}}{\tau_{j_{1}j_{2}}^{t^{\prime }}}. \end{array}$$ (6)

From this we derive the column-(and row-)wise posterior of the matrix **Ω**_*t*_. Without loss of generality we illustrate this for the last column (row). Hereto denote the 2 × 2 block partition of a matrix **X** by

$$\begin{array}{c} X=(\begin{array}{cc} X_{11} & x_{12}\\ x_{12}^{⊤} & x_{22} \end{array}), \end{array}$$ (7)

with **X**_11_ and **x**_12_ a (*p* − 1) × (*p* − 1) and (*p* − 1) × 1 dimensional matrix respectively, while *x*_22_ a scalar. Applying this notation to the matrices involved in the posterior (5), using that by the Schur decomposition it holds that

$$\begin{array}{c} |\Omega_{t}\left|=\right|\Omega_{11}^{t}\left|\right|\omega_{22}^{t}-{\omega_{12}^{t}}^{⊤}{\left(\Omega_{11}^{t}\right)}^{-1}\omega_{12}^{t}|, \end{array}$$

and using the identity

$$\begin{array}{c} \text{tr}\left(S_{t}\Omega_{t}\right)=\text{tr}\left(S_{11}^{t}\Omega_{11}^{t}\right)+2\left({s_{12}^{t}}^{⊤}\omega_{12}^{t}\right)+t_{22}^{t}\omega_{22}^{t}, \end{array}$$

we obtain that under the assumption that $\Omega_{11}^{t}$ is (temporarily) known, the posterior for the last column (row) of the *t*-th precision matrix, $p(\omega_{12}^{t},\omega_{22}^{t}\;|\;Y^{t},\;\Omega_{11}^{t},\;{\left\{\Omega \right\}}_{-t},\;\Lambda ,\;\tau )$, is proportional to

$$\begin{array}{ccc} & & {\left[\omega_{22}^{t}-{\left(\omega_{12}^{t}\right)}^{⊤}{\left(\Omega_{11}^{t}\right)}^{-1}\omega_{12}^{t}\right]}^{n_{t}/2}\\ & & \;\times \;\text{exp}\{-\frac{1}{2}[{\omega_{12}^{t}}^{⊤}D_{a_{12}^{t}}\omega_{12}^{t}+2({s_{12}^{t}}^{⊤}\;-\;{b_{12}^{t}}^{⊤})\omega_{12}^{t}\;+(s_{22}^{t}+\lambda_{t})\omega_{22}^{t}]\}, \end{array}$$

where $D_{a_{12}^{t}}$ is the diagonal matrix with $a_{12}^{t}$ on its diagonal. When followed by the change-of-variables

$$\begin{array}{ccc} \gamma_{t} & = & \omega_{22}^{t}-{\left(\omega_{12}^{t}\right)}^{⊤}{\left(\Omega_{11}^{t}\right)}^{-1}\omega_{12}^{t},\\ \delta_{t} & = & \omega_{12}^{t}, \end{array}$$ (8)

the conditional joint distribution of *δ*_*t*_ and *γ*_*t*_, it can easily be seen that

$$\begin{array}{ccc} \gamma_{t}\;|\;Y^{t},\;\Omega_{11}^{t},\;\Lambda ,\;\tau & ∼ & G[\frac{1}{2}n_{t}+1,\frac{1}{2}\left(s_{22}^{t}+\lambda_{t}\right)],\\ \delta_{t}\;|\;Y^{t},\;\Omega_{11}^{t},\;{\left\{\Omega \right\}}_{-t},\;\Lambda ,\;\tau & ∼ & N_{p-1}\left(-\Sigma_{\delta_{t}}\;\left[{\left(s_{12}^{t}\right)}^{⊤}-{\left(b_{12}^{t}\right)}^{⊤}\right],\Sigma_{\delta_{t}}\right), \end{array}$$ (9)

where $\Sigma_{\delta_{t}}={\left[D_{a_{12}^{t}}+\left(s_{22}^{t}+\lambda_{t}\right){\left(\Omega_{11}^{t}\right)}^{-1}\right]}^{-1}$. Note that the positive definiteness of **Ω**_*t*_ is guaranteed due to that of $\Omega_{11}^{t}$ and the fact that $\gamma_{t}=\omega_{22}^{t}-\omega_{12}^{t_{\prime }}{\left(\Omega_{11}^{t}\right)}^{-1}\omega_{12}^{t}>0$ (cf. [2]).

Next we turn to the scale mixture parameters **τ**_*t*_ corresponding to the priors on the elements of the *t*-th group precision matrix. Gathering terms involving the $\tau_{j_{1},j_{2}}^{t}\text{s}$ we find that, conditionally on **Ω**_*t*_ and *Λ*, they follow an inverse Gaussian distribution for all *j*_1_ < *j*_2_:

$$\begin{array}{ccc} 1/\tau_{j_{1}j_{2}}^{t}\;|\;\Omega_{t},\Lambda \;\; & ∼ & inv-Gauss{\left\{\lambda_{t}^{2}{\left(\omega_{j_{1}j_{2}}^{t}\right)}^{-2}\right]}^{1/2},\lambda_{t}^{2}\},\\ 1/\tau_{j_{1}j_{2}}^{t^{\prime }}\;|\;\Omega_{t},\;\Omega_{t^{\prime }},\;\Lambda & ∼ & inv-Gauss\left\{{\left[\lambda_{t^{\prime },t}^{2}{\left(\omega_{j_{1}j_{2}}^{t}-\omega_{j_{1}j_{2}}^{t^{\prime }}\right)}^{-2}\right]}^{1/2},\lambda_{t^{\prime },t}^{2}\right\},\;\;t^{\prime }\neq t, \end{array}$$ (10)

in which the inverse Gaussian distribution parametrization of [19] is used.

In a similar fashion the posterior conditional distributions of the λ_*t*_ and λ_*t*′, *t*_ can be derived. Based on their gamma priors given in the hierarchical model (4), their full conditional distributions are gamma distributions as well:

$$\begin{array}{ccc} \lambda_{t}\;|\;\Omega_{t} & ∼ & G(r+\frac{1}{2}p\left(p+1\right),\;s+\sum_{j_{1}\leq j_{2}}\left|\omega_{j_{1}j_{2}}^{t}\right|),\\ \lambda_{t^{\prime },t}\;|\;\Omega_{t^{\prime }},\Omega_{t} & ∼ & G(r+\frac{1}{2}p\left(p+1\right),\;s+\sum_{j_{1}\leq j_{2}}\left|\omega_{j_{1}j_{2}}^{t^{\prime }}-\omega_{j_{1}j_{2}}^{t}\right|),\;\;t^{\prime }\neq t. \end{array}$$ (11)

#### 2.1.1 Sampling from the posteriors

The posterior densities derived above facilitate sampling from the joint posterior of the precision matrices. This is achieved as described in Box 1.

**Box 1: Bayesian fused graphical lasso algorithm**

- Initialise **Λ**, ***τ*** and **Ω** from their priors (4).
- For *t* = 1, ‥, *T* do:
  - Calculate **A**_*t*_ and **B**_*t*_ from (6) using the current values of ***τ*** and **Ω**.
  - For column (row) *i* = 1, ‥, *p*,
    1. Block partition **A**_*t*_, **B**_*t*_, **Ω**_*t*_ and **S**_*t*_.
    2. Sample *δ*_*t*_ and *γ*_*t*_ from their posteriors (9).
    3. Update the corresponding column and row of **Ω**_*t*_ using the change of variables (8).
- Sample ***τ*** from the posteriors (10).
- Sample the tuning parameters **Λ** from the posteriors (11).

The algorithm then re-iterates. After a burn-in period, when the samples are seen to be representative for the desired posterior, point estimates for the parameters are obtained from these samples through appropriate summary statistics.

Notice that the above presented Bayesian fused graphical lasso estimation procedure shrinks the elements of the precision matrices towards zero but does not actually set them to zero. Sparsity is achieved by post-hoc estimation via selection based on (quantile-based) Bayesian credible intervals.

### 2.2 Mixture models for multi-group data

The Bayesian fused graphical lasso algorithm presented above can be used to jointly recover graphical networks for multiple data sets. In this subsection we extend the method to the case where the data not only come from multiple groups, but also within each group there may exist multiple sub-populations.

The data stored in the *n* × *p* matrix **Y**, stem from *T* independent *known* groups as before. Additionally, it is assumed that within each group the sample originates from a heterogeneous population. The population of group *t*, *t* = 1, …, *T*, comprises *K*_*t*_ independent *unknown* subgroups. Let, *Z*_*i*_ denote the latent random variable that indicates the *i*-th individual’s subgroup membership, *i* = *n*_<*t*_ + 1, …, *n*_<*t*_ + *n*_*t*_, *t* = 1, …, *T*. In other words, for individual *i* belonging to group *t* we would have *Z*_*i*_ = *k*_*t*_ if this individual would be a member of subgroup *k*_*t*_. With the subgroup information unavailable, the random variable **Y**_*i*_ is assumed to follow the mixture model

$$\begin{array}{ccc} Y_{i} & ∼ & \sum_{k_{t}=1}^{K_{t}}\pi_{t,k_{t}}N_{p}\left(\mu_{t,k_{t}},\Omega_{t,k_{t}}^{-1}\right),\;i=n_{<t}+1,\ldots ,n_{<t}+n_{t},t=1,\ldots ,T \end{array}$$ (12)

with $\pi_{t,k_{t}}=p\left(Z_{i}=k_{t}\right)$ being the probability that individual *i* belonging to group *t* is a member of subgroup *k*_*t*_. Hence, these mixing proportions $\pi_{t,k_{t}}$ sum to one group-wise: $\sum_{k_{t}=1}^{K_{t}}\pi_{t,k_{t}}=1$. Moreover, given the component memberships *Z*_*i*_, the data from each mixture component, corresponding to the subgroup×group-combinations, follow a multivariate Gaussian distribution:

$$\begin{array}{c} Y_{i}\;|\;Z_{i}=k_{t}∼N_{p}\left(\mu_{t,k_{t}},\Omega_{t,k_{t}}^{-1}\right). \end{array}$$

Since data within and across groups are independent, the likelihood L for this situation is given by

$$\begin{array}{ccc} L(Y|Z,\Xi ) & = & \prod_{t=1}^{T}\prod_{i=n_{<t}+1}^{n_{<t}+n_{t}}\sum_{k_{t}=1}^{K_{t}}\pi_{t,k_{t}}\phi_{\mu_{t,k_{t}},\Omega_{t,k_{t}}^{-1}}\left(Y_{i}\right). \end{array}$$ (13)

Here **Z** = (*Z*_1_, …, *Z*_*n*_), $\Xi =\{\pi_{t,k_{t}},\mu_{t,k_{t}},\Omega_{t,k_{t}}^{-1}:i=n_{<t}+1,\ldots ,n_{<t}+n_{t},t=1,\ldots ,T\}$, the set of all model parameters, and *ϕ*_*a*,*B*_ denotes the density function of the multivariate normal distribution with mean vector **a** and covariance matrix **B**.

Estimation of the mixture model (13) is carried out by first clustering the data points within each group. This is achieved by adopting the Bayesian clustering scheme that assigns informative priors on the component memberships as proposed in [16], and briefly described in Section 2.2.1. Next, the mixture parameters are estimated component-wise. The estimation procedure for the component-wise estimation of the mean parameters is described in Section 2.2.2. The precision matrices are estimated in either of the following two ways:

(a) Bayesian stage-wise (BS), this is separately in a group-wise manner by Gibbs sampling for a single Bayesian graphical lasso as in [7], if there is no reason to assume that the data across groups have a common structure;
(b) jointly with the Bayesian fused (BF) estimation procedure described in Section 2.1 above, if the data across groups are likely to have a common structure.

The corresponding mixture models are called Bayesian stage-wise mixture model (BSM) and Bayesian fused mixture model (BFM), respectively.

#### 2.2.1 Estimation of component memberships

Data clustering is an important step in estimation of mixture models. To improve the estimation procedure, one may consider making use of available additional information such as cluster information or similarity measurements from other data sources. The proposed data allocation strategy, Data Integrative Chinese Restaurant Process (DI-CRP), is a generalization of the Chinese restaurant process (CRP) with additional flexibility that facilitates incorporating external sample-level information in mixture modeling with an unknown number of components [16]. Like CRP, DI-CRP is a data allocation strategy that explores the conditional distribution of the component membership of one data point given that of the rest of data points. This allows the number of mixture components to be determined adaptively. Let $S^{t}={\left(s_{ii^{\prime }}^{t}\right)}_{i\leq i^{\prime }=1}^{n_{t}}$ represent the additional information on similarity of data points in group *t*.

As the data allocation is independently carried out for every group, we will now drop the group index *t* in the notation and denote the index of the *k*_*t*_-th mixture component simply by *k*. We assume the following conditional probabilities for the component membership variables:

$$\begin{array}{c} P\left(Z_{i}=k|Z_{-i},\alpha ,S\right)\propto \{\begin{array}{cc} n_{-i,k}^{*}h_{i}\left(k\right) & \text{if}\;k\;\text{is}\;\text{an}\;\text{existing}\;\text{component}\\ \alpha & \text{if}\;k\;\text{is}\;\text{a}\;\text{new}\;\text{component} \end{array} \end{array}$$ (14)

where *h*_*i*_(*k*) is a function that indicates the overall similarity of the data point *i* with all other data points in component *k*. This function can appear in different forms. Here we use the simple form

$$\begin{array}{c} h_{i}\left(k\right)=1+\sum_{i\neq i^{\prime }}s_{ii^{\prime }}I_{\left\{Z_{i^{\prime }}=k\right\}}, \end{array}$$

and

$$\begin{array}{c} n_{-i,k}^{*}=\sum_{i\neq i^{\prime }}I_{\left\{s_{ii^{\prime }}\geq T_{i}\right\}}I_{\left\{Z_{i^{\prime }}=k\right\}} \end{array}$$

with *I*_{*s*_*ii*′_ ≥ *T*_*i*_}_ as the factor that indicates when a data point is considered to be similar to the rest of data points in a certain component. For example, in our application *T*_*i*_ is assumed to be the third quantile of the similarity values between data point *i* and the rest of the group-specific data points. The reason to introduce $n_{-i,k}^{*}$ is to diminish the influence of a minority of data points in a cluster that have possibly very large similarity values with a new data point. In other words, this criterion is to direct the clustering in such a way that a new data point becomes more likely to end up being clustered in a component of which the majority of the data points shares high similarities with the new data point.

Multiplying the likelihood (12) and the prior (14) we see that the posterior of the latent variables satisfies

$$\begin{array}{c} \begin{array}{cc} P(Z_{i}=k| & Y_{i},Z_{-i},\mu_{k},\Omega_{k},\alpha ,S)\\ & \propto \{\begin{array}{c} n_{-i,k}^{*}h\left(c_{i},k\right)\;p\left(Y_{i}\;|\;\mu_{k},\;\Omega_{k}\right),\;\text{if}\;k\;\text{is}\;\text{an}\;\text{already}\;\text{existing}\;\text{component,}\\ \alpha \int p\left(Y_{i}\;|\;\mu_{k},\;\Omega_{k}\right)p\left(\mu_{k},\Omega_{k}\right)d\mu_{k}d\Omega_{k},\;\text{if}\;k\;\text{is}\;\text{a}\;\text{new}\;\text{component.} \end{array} \end{array} \end{array}$$ (15)

As the integral in (15) is not analytically tractable, it can be approximated by Monte Carlo samples as described in [16].

The number of mixture components *K* is largely controlled by the choice of *α*, in that larger values lead to more components. Following [20], we assume a gamma prior with mean *a* and rate parameter *b* for the concentration parameter *α*. The full conditional distribution can be derived given the number of components *K*—which is implied by the fact that **Z** is given—following the hierarchy

$$\begin{array}{ccc} \alpha |\zeta ,K & ∼ & \rho_{\zeta }\;G\;\left(a+K,b-log\left(\zeta \right)\right)+\left(1-\rho_{\zeta }\right)\;G\;\left(\alpha +K-1,b-log\left(\zeta \right)\right),\\ \zeta |\alpha & ∼ & B(\alpha +1,n), \end{array}$$ (16)

where $\frac{\rho_{\zeta }}{1-\rho_{\zeta }}=\frac{a+K-1}{n(b-log(\zeta ))}$.

The data allocation probabilities above form the basis for building a clustering algorithm that functions through sampling from posterior probabilities of component memberships.

#### 2.2.2 Estimation of component means

Once the data in all groups are clustered, i.e. **Z** is known, the component-specific parameters are to be updated. In contrast to the previous sections, here the component-specific mean parameters are assumed unknown with a conditional prior distribution (again dropping group indices)

$$\begin{array}{c} \mu_{k}|\;\Omega_{k}∼N_{p}\left(\mu_{\text{0}},\kappa_{\text{0}}^{-1}\Omega_{k}\right),\;k=1,\ldots ,K, \end{array}$$ (17)

with ***μ***_*k*_ and **Ω**_*k*_ the *k*-th component mixture mean and precision parameters. In this prior distribution ***μ***_0_ and *κ*_0_ are hyperparameters of vector and scalar type, respectively, and they are the same for all subgroups over all groups. The prior distribution (17) is conjugate and, combined with the data likelihood, yields the posterior

$$\begin{array}{c} \mu_{k}\;|\;Y,\Omega_{k}∼N_{p}\left(m_{k},{\left(n_{k}+\kappa_{\text{0}}\right)}^{-1}\Omega_{k}\right),\;k=1,\ldots ,K, \end{array}$$ (18)

with *n*_*k*_ the number of data points assigned to mixture component *k* and

$$\begin{array}{c} m_{k}=\frac{n_{k}}{n_{k}+\kappa_{\text{0}}}{\bar{Y}}_{k}+\frac{\kappa_{\text{0}}}{n_{k}+\kappa_{\text{0}}}\mu_{\text{0}}, \end{array}$$ (19)

where ${\bar{Y}}_{k}$ is the *p*-dimensional mean of the data vectors assigned to the *k*-th group. We note that we do not enforce sparsity on the component mean parameters. With a different prior setting, this could also be established.

#### 2.2.3 Algorithm

The algorithm for computing component memberships is given in Box 2. Note that for simplicity the component indices are denoted by *k* or *k*′, instead of *t*, *k*_*t*_ or *t*, *k*′_*t*_.

**Box 2: MCMC algorithm for Bayesian groupwise mixture (BSM) and Bayesian fused mixture (BFM) methods**

1. Update number of components:
  for *i* = *n*_<*t*−1_ + 1, …, *n*_<*t*−1_ + *n*_*t*_, given a current clustering *Z*_*i*_ = *k*, if *i* is not a singleton data point, create a new component *k*′ by sampling from the prior distributions of ***μ*** and **Ω**, and update *Z*_*i*_ = *k*′ with probability
  $$\begin{array}{c} min\{1,\frac{\alpha }{\left(n_{t}-1\right)}\times \frac{\phi_{\mu_{k^{\prime }},\Omega_{k^{\prime }}^{-1}}\left(Y_{i}\right)}{\phi_{\mu_{k},\Omega_{k}^{-1}}\left(Y_{i}\right)}\}, \end{array}$$
  and if *i* is the only data point in component *k* (singleton), propose *k*′ among already existing components with a probability proportional to $n_{-i,k^{\prime }}^{⋆}$, and update *z*_*i*_ = *k*′ with probability
  $$\begin{array}{c} min\{1,\frac{n_{t}-1}{\alpha }\times \frac{h_{i}\left(k^{\prime }\right)\phi_{\mu_{k^{\prime }},\Omega_{k^{\prime }}^{-1}}\left(Y_{i}\right)}{h_{i}\left(k\right)\phi_{\mu_{k},\Omega_{k}^{-1}}\left(Y_{i}\right)}\}. \end{array}$$
2. Update component memberships:
  for *i* = *n*_<*t*−1_+ 1, …, *n*_<*t*−1_ + *n*_*t*_, if data point *i* belongs to a component with more than one occupant, update its component membership with probability equal to
  $$\begin{array}{c} \frac{n_{-i,k}^{⋆}h_{i}\left(k\right)\;\phi_{\mu_{k},\Omega_{k}^{-1}}\left(Y_{i}\right)}{\sum_{k=1}^{K}n_{-i,k}^{⋆}h_{i}\left(k\right)\;\phi_{\mu_{k},\Omega_{k}^{-1}}\left(Y_{i}\right),} \end{array}$$
  otherwise do nothing.
3. Update mixture parameters:
  3.1 update mixture means from posterior (18) and (19).
  3.2 based on the application choose either BMS or BFM and
    **BSM**: update precision matrices corresponding to the current group by Gibbs sampling for a single Bayesian graphical lasso as in [7].
    **BFM**: repeat steps 1–2 and 3.1 for all groups, then jointly update all precision matrices from all sub-groups by the Gibbs sampling procedure in Box 1 of Section 2.1.1.
4. Iteration:
  repeat steps 1–4 until convergence.
  The software package that implements the algorithm and illustrative examples are publicly available from https://github.com/mehranaflak/IMLR_TextGGN

To initialize clustering, we fix the maximum number of mixture components to *K*_*max*_ (≤*n*). The first component is created by sampling parameter values from their prior distributions. Next, a data point having the largest normal density value among all data points is assigned to this component. The second component is created in the same way as the first one, but, the second data point can be assigned to either the first component or to the second one based on the maximum value of the generated density values. This continues until all data points are assigned to a finite number *K* (≤*K*_*max*_) of components.

One sweep of the algorithm updates the number of components, the component memberships and the component-wise parameters. At each iteration of the Metropolis algorithm the updated component memberships are the basis of the clustering, followed by inference of the component-specific mean and precision parameters. We use the posterior means to estimate the component specific parameters. As the estimate for the number of components *K*, we use the final number of components after the algorithm has converged.

Notice that in general the MCMC algorithm starts with multiple (known) groups and explores the structure of data within each group in order to further cluster them into smaller sub-groups (i.e. steps 1–2). Also notice the difference between step 1 and step 2 of the algorithm. While step 1 of the algorithm controls the birth of a new component or death of an existing component, step 2 attempts to update the clustering by exchanging data points.

## 3 Results

### 3.1 Simulation

The performance of the proposed methods was assessed via a simulation analysis with three objectives: i) evaluation of the performances of the two approaches BS and BF in recovering graphical networks corresponding to multiple data sets, ii) comparison of the performance of BSM and BFM in the proposed mixture context, and iii) assessment of the accuracy of the cluster assignment scheme DICRP and its comparison to that of the original CRP in the present context. Two simulation studies were conducted as described below.

**Fig 1:**
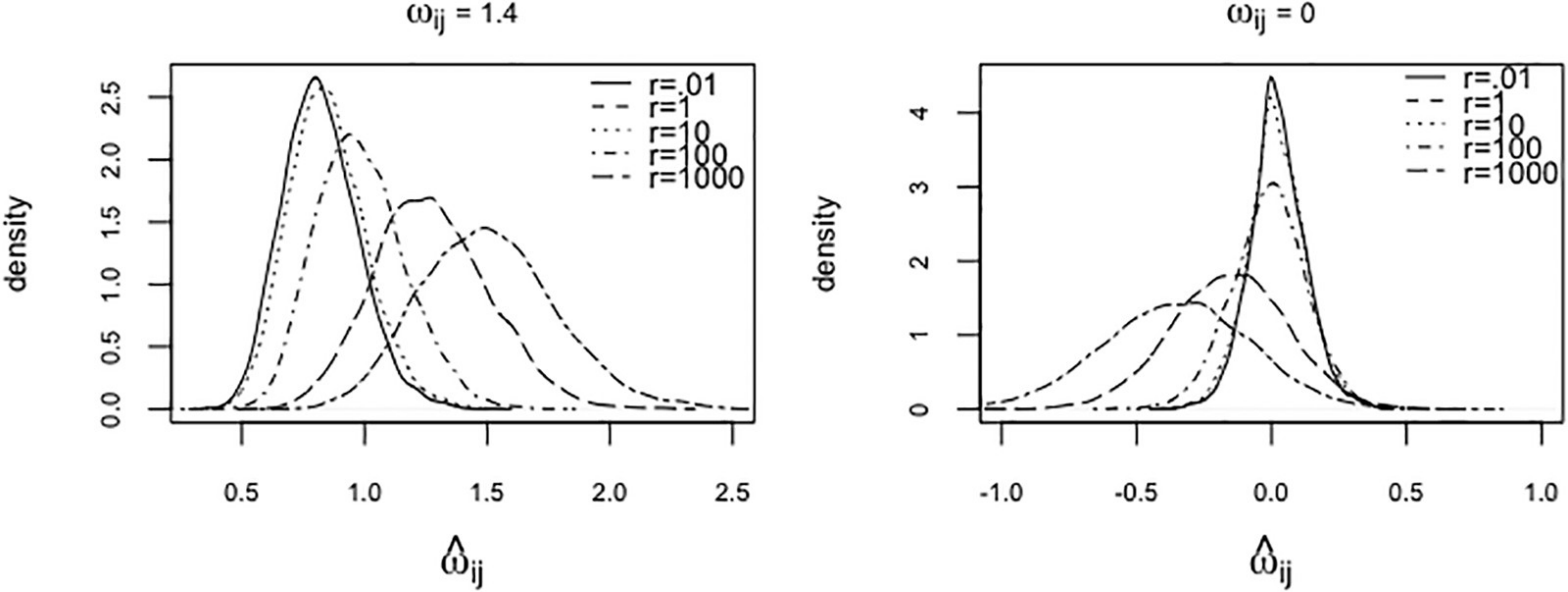
Element-wise empirical posterior distribution of precision matrices. Empirical posterior density by varying tuning parameter for zero (left) and non-zero (right) element of **Ω** for *p* = 10.

The hyper-parameter values were assigned mainly based on previous studies and partly based on independent simulations. Firstly, the rate parameter *s* controlling the tuning parameters has to be sufficiently larger than zero to avoid computational issues, therefore it was set to unity (see [17] for a substantiation of this choice). We took the shape parameter *r* = 0.001 based on a simulation study. Fig 1 illustrates the impact of the shape parameter on the empirical posterior density of zero and non-zero elements of the precision matrix. Secondly, hyperparameters of the concentration parameter *α* were set to *a* = *b* = 1 as recommended in [21] and [22]. Lastly, the hyperparameters corresponding to the mixture means ***μ***_0_, *κ*_0_ were set to a zero vector of dimension *p* and a scalar equal to unity, respectively. The reason for assigning a less informative prior to the mixture means is that our main objective of this study which is to focus on the estimation of the precision matrices.

**Fig 2:**
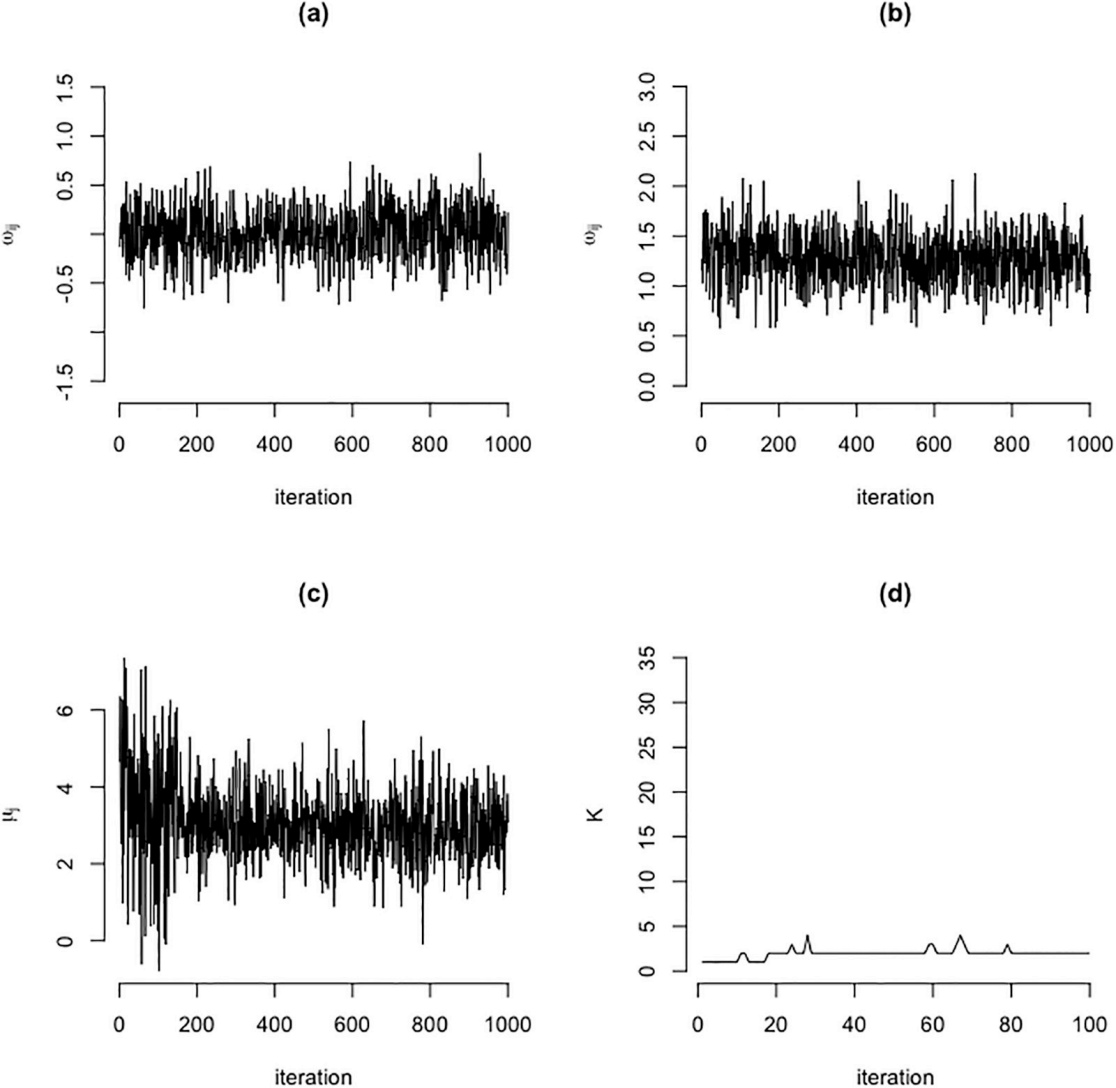
Mixing of MCMC samples. Trace of Gibbs samples of zero(a) and non-zero (b) element of precision matrix, mixture mean (c), and the number of components (d).

The MCMC algorithm used 1000 Gibbs iterations and a burn-in period of 100 iterations. These numbers of iteration were motivated from preliminary simulation studies (not shown) in which we noticed that the MCMC algorithm for a similar problem appeared to have reached convergence for significantly fewer (≈50) iterations. In our applications, after a burn-in period of 100 iterations the samples were seen to be representative for the desired posterior, see Fig 2 which shows one example of posterior sample traces from zero and non-zero elements of the ground-truth inverse covariance parameters as an illustration that there was a good mixing around the true parameter values with no particular pattern.

Point estimates of the parameters were obtained by posterior mean calculation over the iterations. Sparsification was carried out through construction of the 95% two-sided Bayesian credible intervals. Then, the presence of an edge was inferred when the corresponding credible interval did not contain zero. The performance of the estimation of the model parameters was measured by Frobenius loss, for both the mean vector and the iprecision matrices. The ability to reconstruct the underling conditional independence graph was evaluated by means of the F-score (i.e. the harmonic mean of precision and sensitivity).

**Fig 3:**
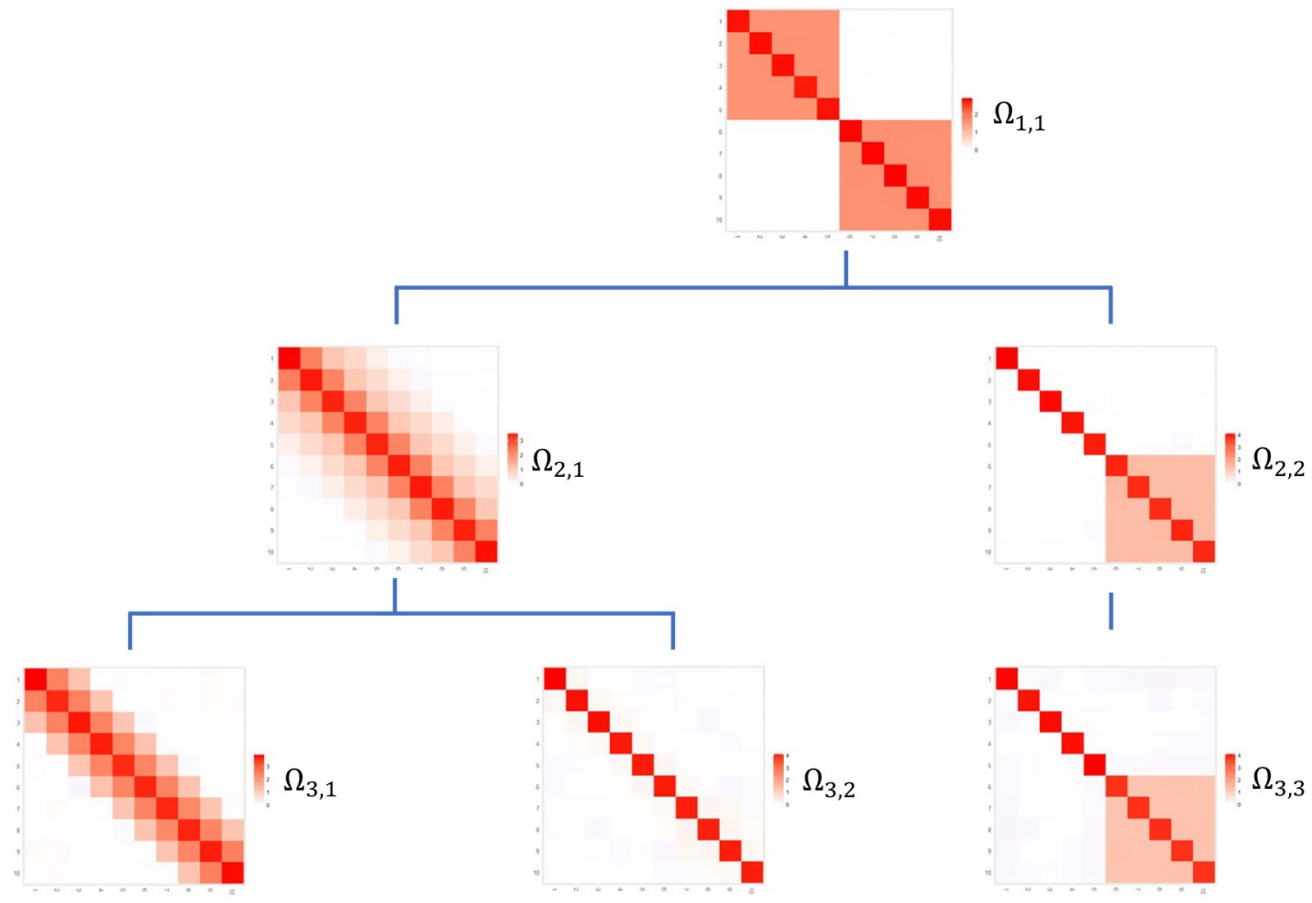
Structure of precision matrices. Graphical network structure for a dataset with 3 stages that was used for data generation.

Several settings were fixed for both simulation studies. In the first simulation study the BS and BF methods were compared with respect to their ability to estimate various (six) precision matrices. These matrices either had conditional independence graphs with similar structures as depicted in Fig 3, or were randomly generated positive definite matrices without any imposed similarity constraints. Both cases defined six *p*-variate Gaussian graphical models. All models had zero mean vectors. For each case 100 data sets of size *n* were generated from each of the six *p*-variate Gaussian models. This amounted to 600 simulated data vectors in each case. This was repeated for several combinations of sample sizes and dimensions, *n* ∈ {50, 100, 500} and *p* ∈ {5, 10, 20, 30}. The simulated data were analyzed by both BF and BS methods in order to derive estimates of the precision matrices.

**Fig 4:**
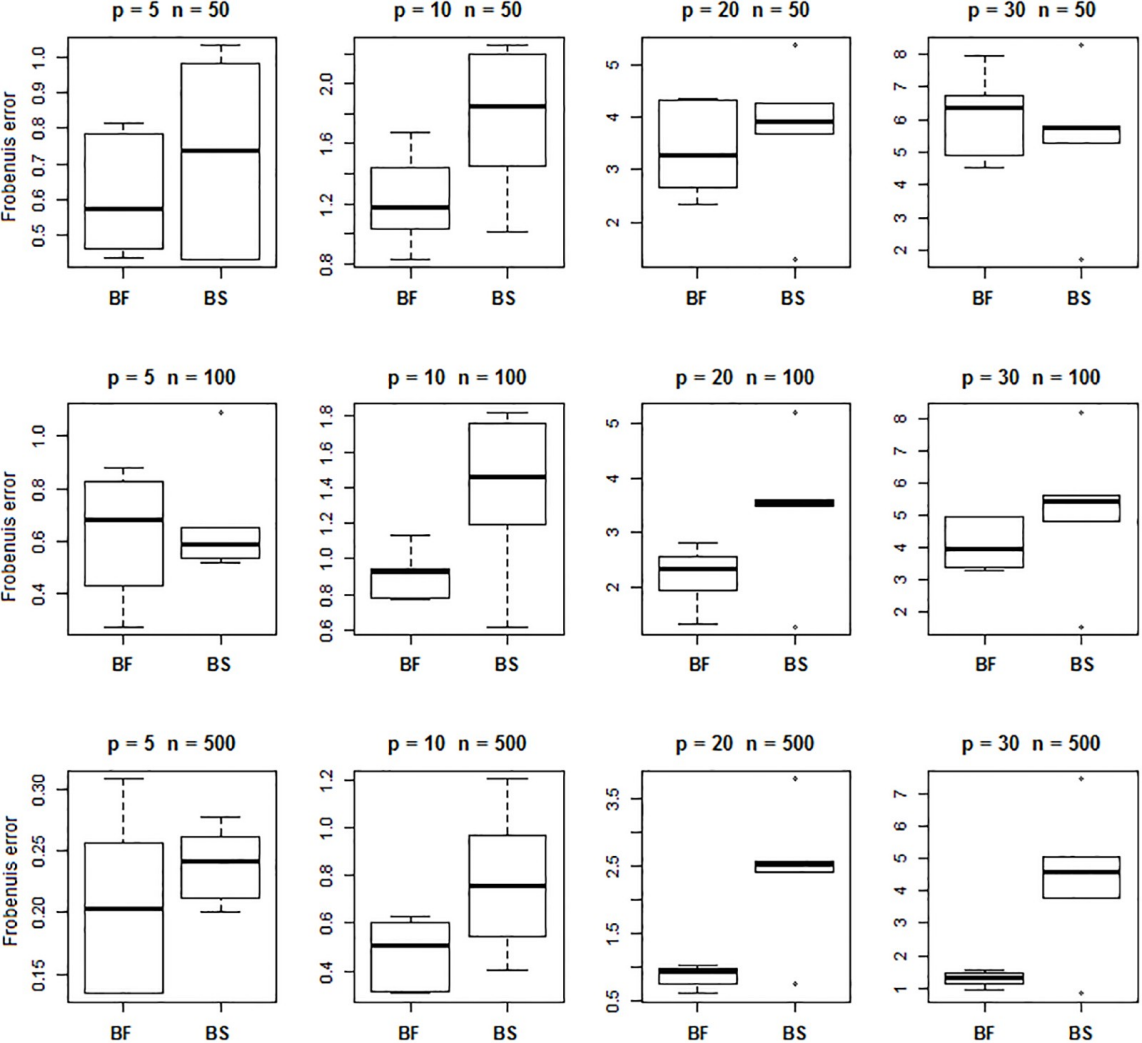
Prediction loss of precision matrices. Frobenuis errors for estimation of precision matrices with a defined *shared* structure by BF and BS methods.

**Fig 5:**
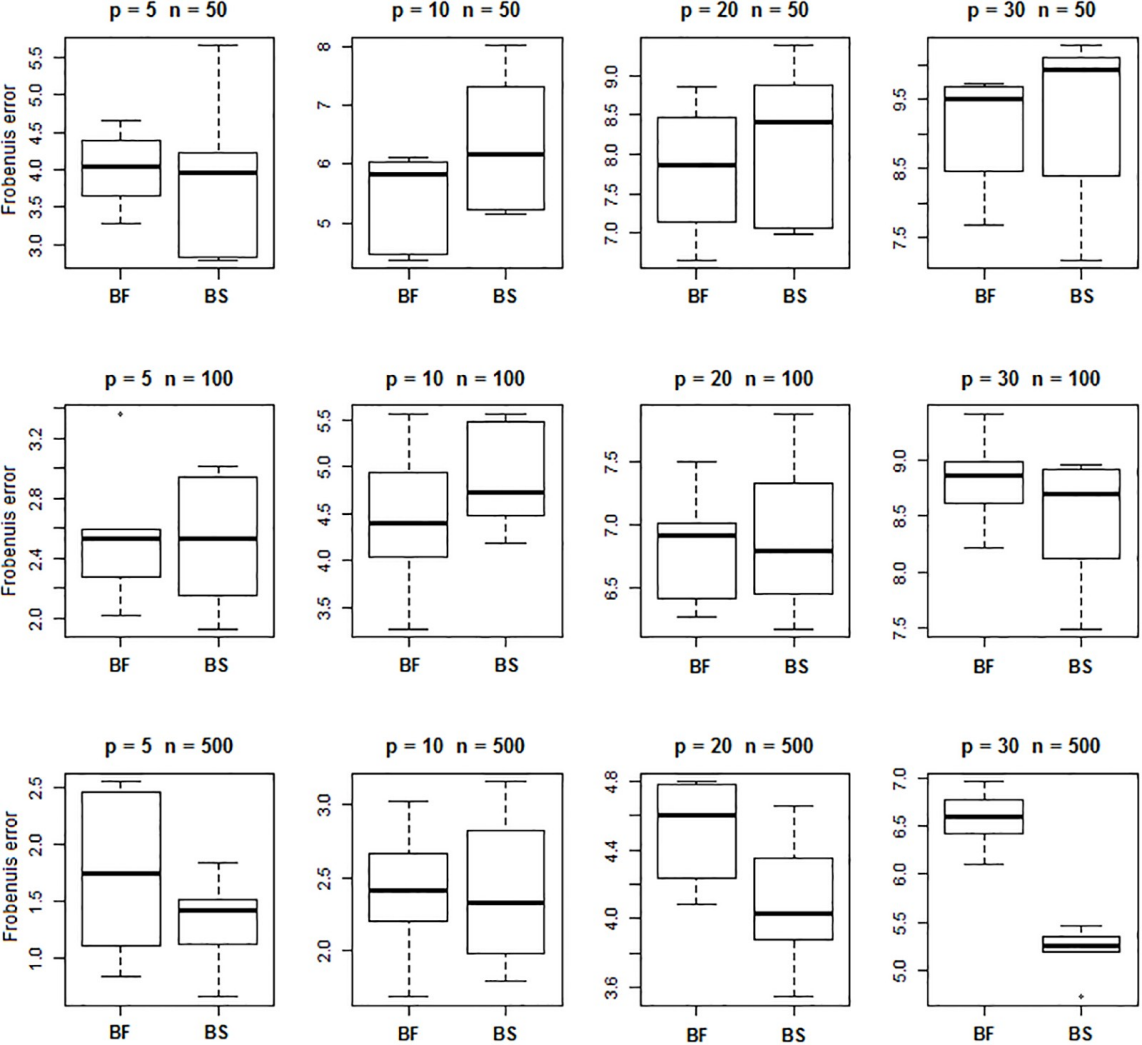
Prediction loss of precision parameter estimation. Frobenuis errors for estimation of precision matrices with *random* structure by BF and BS methods.

The performance of BS and BF was measured based on average squared estimation errors over the 100 simulated data vectors and over all 6 precision matrices. Figs 4 and 5 present boxplots of the estimation errors for the different combinations of sample sizes and dimensions. From Fig 4 it is evident that the BF procedure yields smaller Frobenius error than the BS procedure for precision matrices with similar conditional independence graphs. For the randomly generated networks without similarity structure, the two methods produce similar results, see Fig 5.

In the second simulation study the performance of the BSM and BFM procedures for the estimation of a mixture of Gaussian graphical models, as given by (12), were assessed. The hyper-parameters regarding the precision matrices were selected as above, and we set ***μ***_0_ = 0 and *κ*_0_ = 1 for the sake of simplicity. The simulation study considered three consecutive time periods. For each period data were generated from (12) such that the data exhibited more heterogeneity in subsequent periods: the data in the first period were homogeneous, whereas data of the second and third period stemmed from two and three sub-populations, respectively. The conditional independence graphs associated with the precision matrices were topologically related to mimic a simple evolution as in Fig 3. The data were generated according to the parameter settings in the Supporting Information with varying sample sizes *n* ∈ {100, 200, 300, 400, 500, 1000} and dimensions *p* ∈ {10, 20, 30, 40} with 50 independent data sets for each (*n*, *p*) combination. We employed BSM and BFM methods on all data sets to fit mixture models.

**Fig 6:**
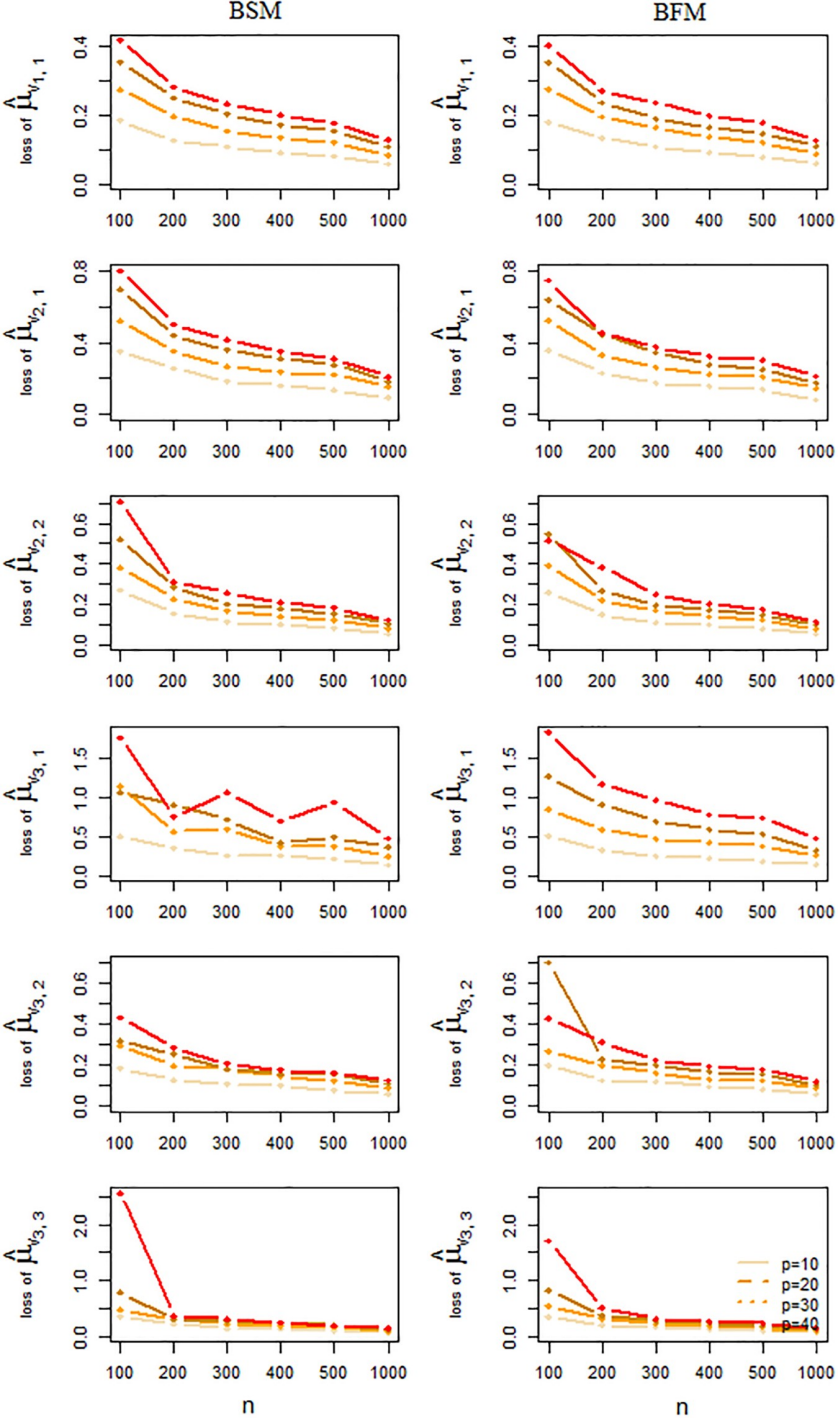
Prediction loss of component-wise mean parameter estimation. Frobenuis errors of mixture mean estimation averaged over 50 independent simulated datasets by BSM and BFM methods.

**Fig 7:**
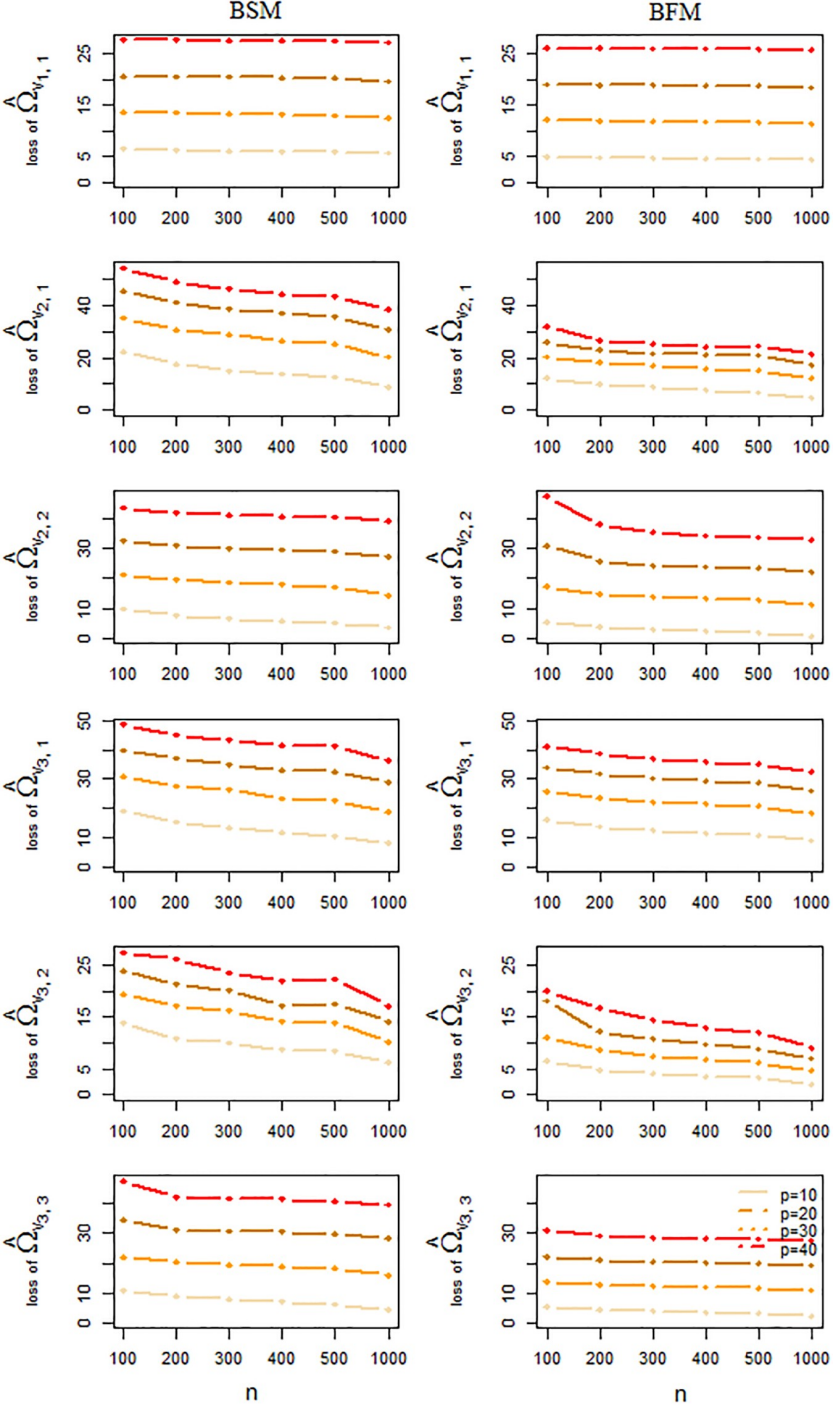
Prediction loss of component-wise precision parameter estimation. Frobenuis errors of precision parameter estimation averaged over 50 independent simulated datasets by BSM and BFM methods.

**Fig 8:**
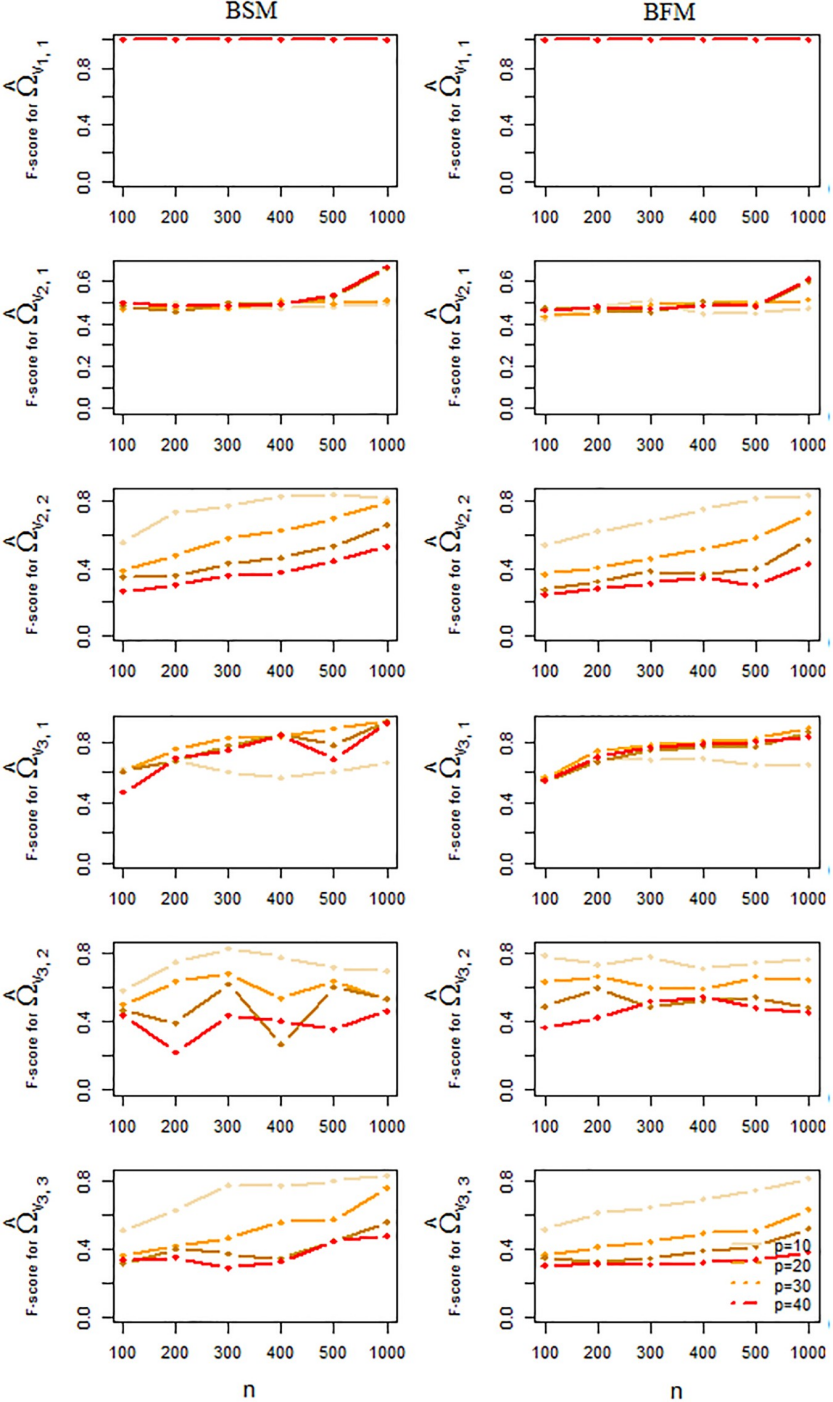
Classification accuracy. F-scores corresponding to estimation of sparsified precision matrices averaged over 50 independent simulated datasets by BSM and BFM.

The performance of the BSM and BFM methods for the estimation of the mixture means and precision matrices, as well as the quality of the edge presence/absence classification as obtained from this simulation study is summarized in Figs 6 and 7. Each point in these plots represents an average taken over 50 independent results. From these Figs we conclude that a larger sample size generally tends to increase the accuracy of the estimation and to alleviate estimation errors. Larger data dimensions, on the other hand, have a reverse influence on accuracy of the results. With respect to precision matrix estimation Fig 7 suggests a slightly better performance for the BFM method over the BSM one, especially for higher dimensions. But for mixture mean estimation or graph recovery there is no strong evidence of superiority for any of the two methods, see Figs 6 and 8.

The influence of the proposed component membership priors, as specified in (15), on the cluster assignment was assessed. The clustering approaches were compared for BSM only, as the clustering in both the BSM and BFM procedures is carried out for each period separately. The data comprising six subgroups were drawn from the mixture model (12) with the settings as specified in the Supporting Information, assuming various sample sizes and dimensions: *n* ∈ {50, 100, 300} and *p* ∈ {5, 10, 16, 20}. For each (*n*, *p*) combination 50 independent data sets were generated. For the assessment two different scenarios were considered: *a)* no additional information on the samples is available: BSM-CRP, and *b)* external evidence on the samples’ similarity is available: BSM-DICRP. In scenario *a)* the priors (15) were equivalent to those of CRP as S=0. In scenario *b)* priors (15) were used with a non-zero similarity matrix. For the data in each stage, the similarity matrix S was generated based on the true clustering of the data points, where *s*_*ij*_ = 1 if data point *i* lies in the same cluster as data point *j*, and *s*_*ij*_ = 0 otherwise. These methods were applied to all of the simulated data sets to measure the performance of the clustering scheme when additional clustering information is available.

**Fig 9:**
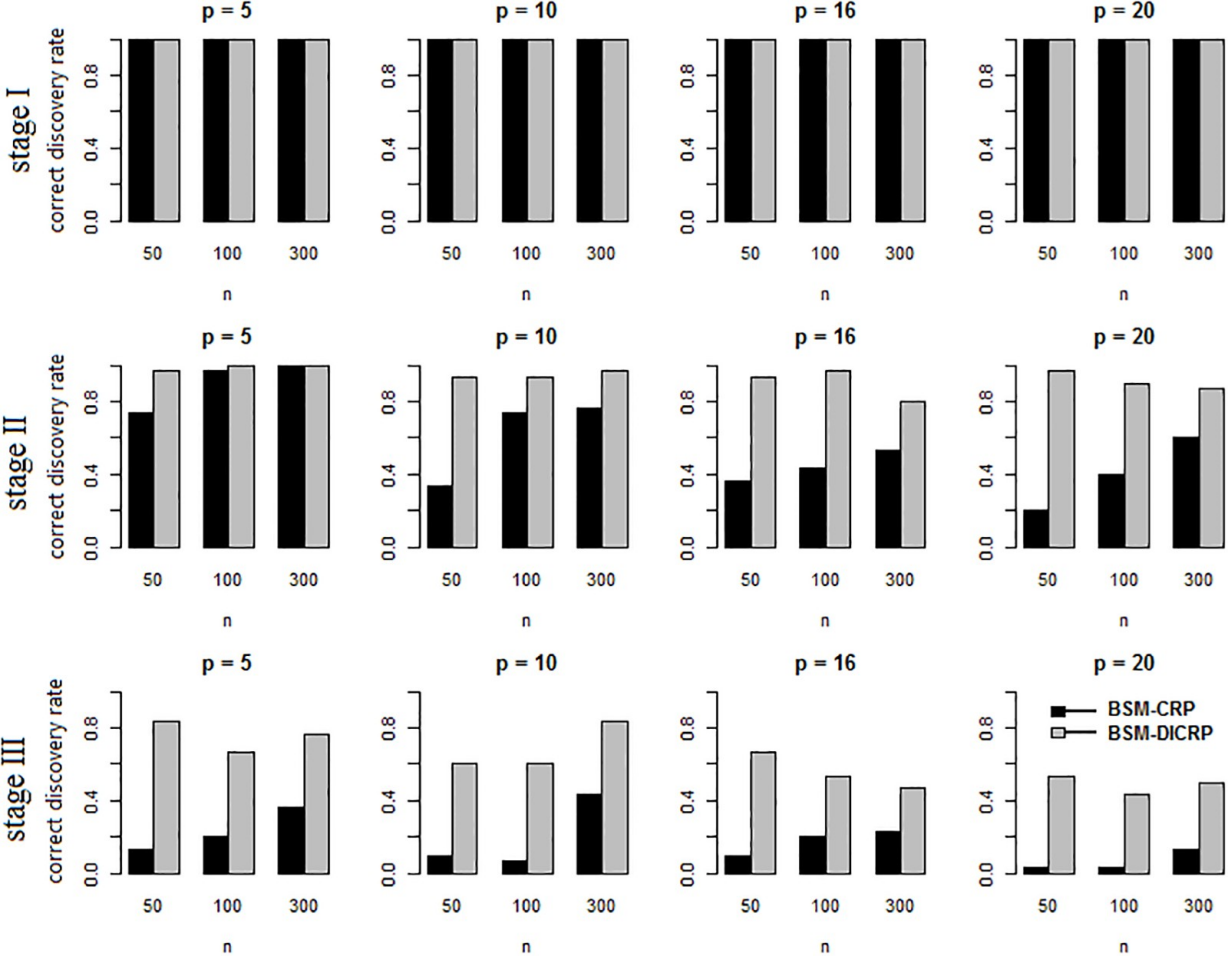
Mixture clustering accuracy. Estimation of number of components in a 3-stage simulated dataset with number of components *K*_1_ = 1, *K*_2_ = 2 and *K*_3_ = 3.

Next, the clustering performance is measured by the proportion of correctly identified ground-truth mixture clusters. The results are summarized in Fig 9 where CRP or DICRP are compared. For example, the top row of this figure shows that in 100% of the simulations the estimated number of components is equal to the ground-truth number of components (= 1). Generally, these results show that, when additional clustering information is available, BSM combined with DICRP yields a better (clustering) performance than BSM combined with the CRP prior on the component memberships.

**Fig 10:**
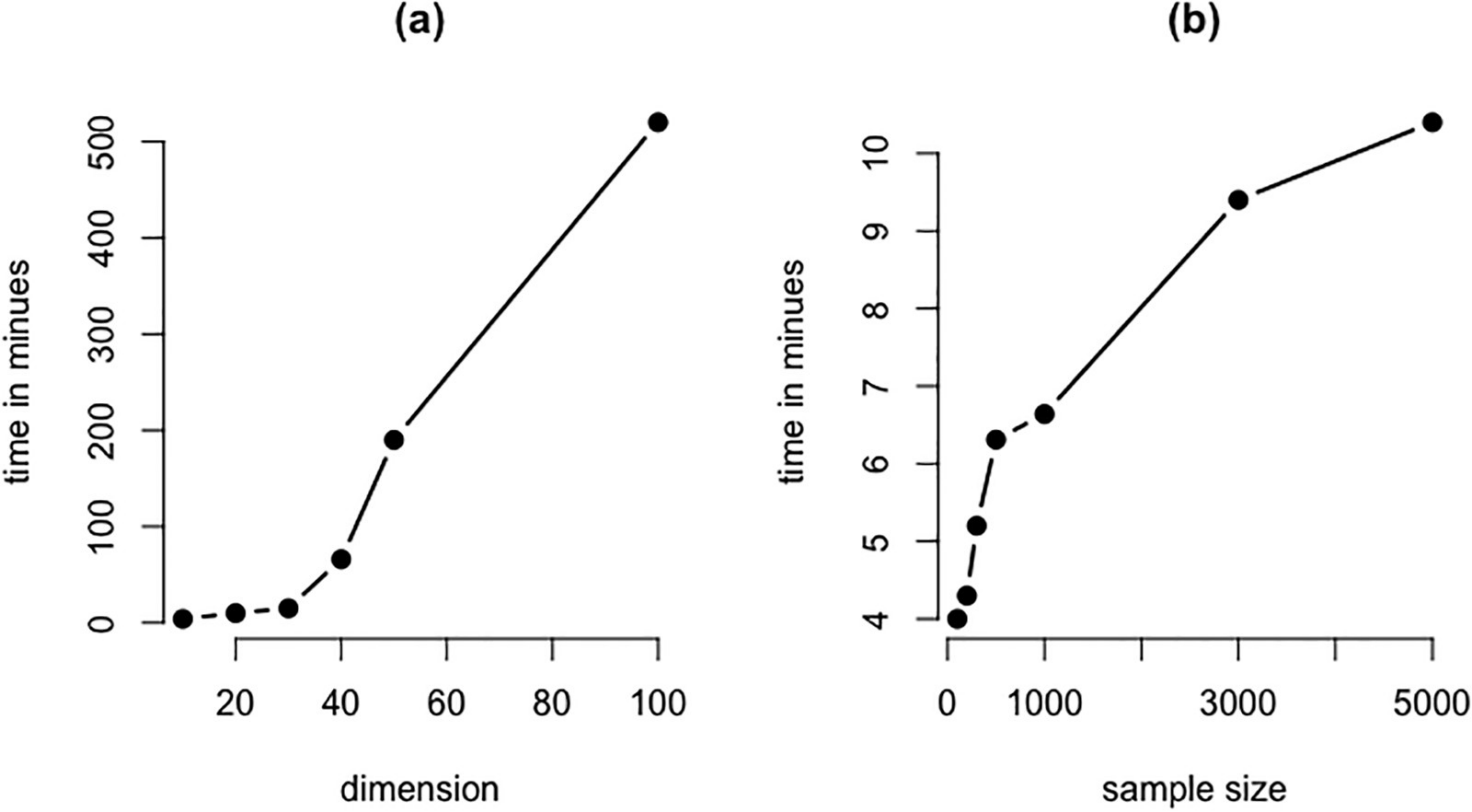
Computational time. Computational time to estimate mixture graphical networks as a function of (a) the data dimension with a fixed sample size of 100 and (b) the sample size with a fixed dimension of 10.

Finally, we measured the computational time of the algorithm in order to illustrate the scalability of our approach. The results are shown only for the BFM approach using the abovementioned simulation settings for varying data dimension and sample size. In summary, as depicted in Fig 10, despite using an efficient block sampling technique the scalabilty becomes an issue for higher dimensions, while this is not the case for increasing number of samples.

### 3.2 Analysis of Twitter data

In this section, we illustrate an example on how to summarize Twitter data into networks of terms or ‘words’, using the BSM and BSF methods. The inferred conditional independence graphs are then studied to signify topics and their evolution through time. To this end, we analyzed tweets regarding the Iranian 2009 presidential election.

#### 3.2.1 Context

The two main candidates of the Iranian presidential election of June 2009 were Mir-Hossein Mousavi of the reformist “Green Movement” and president Mahmoud Ahmadi-Nejad, running for a new term. The latter won the election, but the result was disputed by alleged voting irregularities and fraud. These allegations gave rise to mass protests by supportors of Mir-Hossein Mousavi’s “Green Movement” against the president-elect.

A time line of events crucial to the 2009 Iranian presidential election is summarized below for three consecutive periods. Words in italic are most frequently used in the tweets of the three time periods.

**Period I**

*2009-05-12* Election day.
*2009-05-15* First mass rally to *protest* against the election results with demonstrators chanting words like ‘*Allaho Akbar*’.
*2009-05-19* Speech by *Khamenei*, the supreme leader, on *Friday*’s (weekend day in Iran) prayer.
*2009-05-20* *Neda* (a *Mousavi*-supporter was *shot* and many more supporters were imprisoned at the *Evin prison*. *human rights* groups and the president of the United States issued statements urging the end to violence against protesters.
*2009-06-09* Demonstrations on the tenth anniversary of the 18-Tir uprising. This refers to the Iranian Student Protests of July 1999 (also known as 18th of Tir (7–13 July) and Kuye Daneshgah Disaster in Iran) were the most widespread and violent public protests to occur in Iran since the early years of the Iranian Revolution. During the 18-Tir uprising students asked for greater freedom of speech, among others.
*2009-06-17* Friday’s prayer sermon by *Akbar Rafsanjani* attended by ≥ two million people, including the leaders of the *“Green Movement”*. Among the people that were *arrested* and taken to the *Evin prison* was *Shadi Sadr*, an Iranian women rights activist who featured prominently in the news.
*2009-08-01* Start of the trial against the people arrested in the protests. These trials were condemned by the protesters associated with the *“Green Movement”*.
*2009-09-18* On Friday Quds day, an annual event held on the last Friday of the Ramadan that was initiated by the Islamic Republic of Iran in 1979, the second wave of major protests in Tehran and other cities took place.

**Period II**

*2009-11-04* Students’ day, which refers to the day that the USA embassy was conquered in Iran (November 04, 1379), saw another large demonstration with people chanting among others slogans like “*Allaho Akbar*”, “A green Iran doesn’t need *nuclear weapons*”, and “*death* to dictator”, among others. On the same day the reformist candidate *Mousavi* was grounded and could no longer leave house.
*2009-12-07* Iranian scholar day, which is the anniversary of the murder of three students of University of Tehran on December 7, 1953 by Iranian police., show thousands of students protesting against the government and demanding a regime change. In a speach *Akbar Rafsanjani* criticized the strict measures taken by the Iranian government.
*2009-12-19* Ayattollah Ali *Montazeri*, an influential figure of the *“Green Movement”*, died.
*2009-12-21* A rally took place on the occasion of the funeral ceremony for Ayatollah *Montazeri*. Simultaneously, reports were published claiming *torture* and rape of prisoners associated with the *“Green Movement”*. The clashes between supporters of the ‘*“Green Movement”* and the police continued for a few days in the cities of Isfahan and Qom.
*2009-12-27* At the day of “Ashura” another big demonstration took place during which many people were shot and killed, including Seyed Ali *Mousavi*, the nephew of Mir-Hossen Mousavi. Ashura is the tenth day of Muharram in the Islamic calendar, and commemorates the death of Husayn ibn Ali, the grandson of Muhammad. For a majority of Shia’s Muslim Ashura has become a ceremonial mourning day.
*2009-12-28* Western countries condemned the violations of the protesters’ *human rights* by the Iranian government.
*2010-02-17* Signing of a petition by supporters of the *“Green Movement”* against a law that would limit *women’s rights*.

**Period III**

*2010-05-10* For fear of violence by the leaders of the *“Green Movement”*, the planned demonstrations on the first anniversary of the disputed election was cancelled. Police violence against women who allegedly were improperly clothed in public.
*2010-05-11* Citizens were warned of the probable consequences of participation in the anniversary of the election.
*2010-05-12* Minor demonstrations took place, but ended prematurely due to governmental interference.

#### 3.2.2 Data

**Fig 11:**
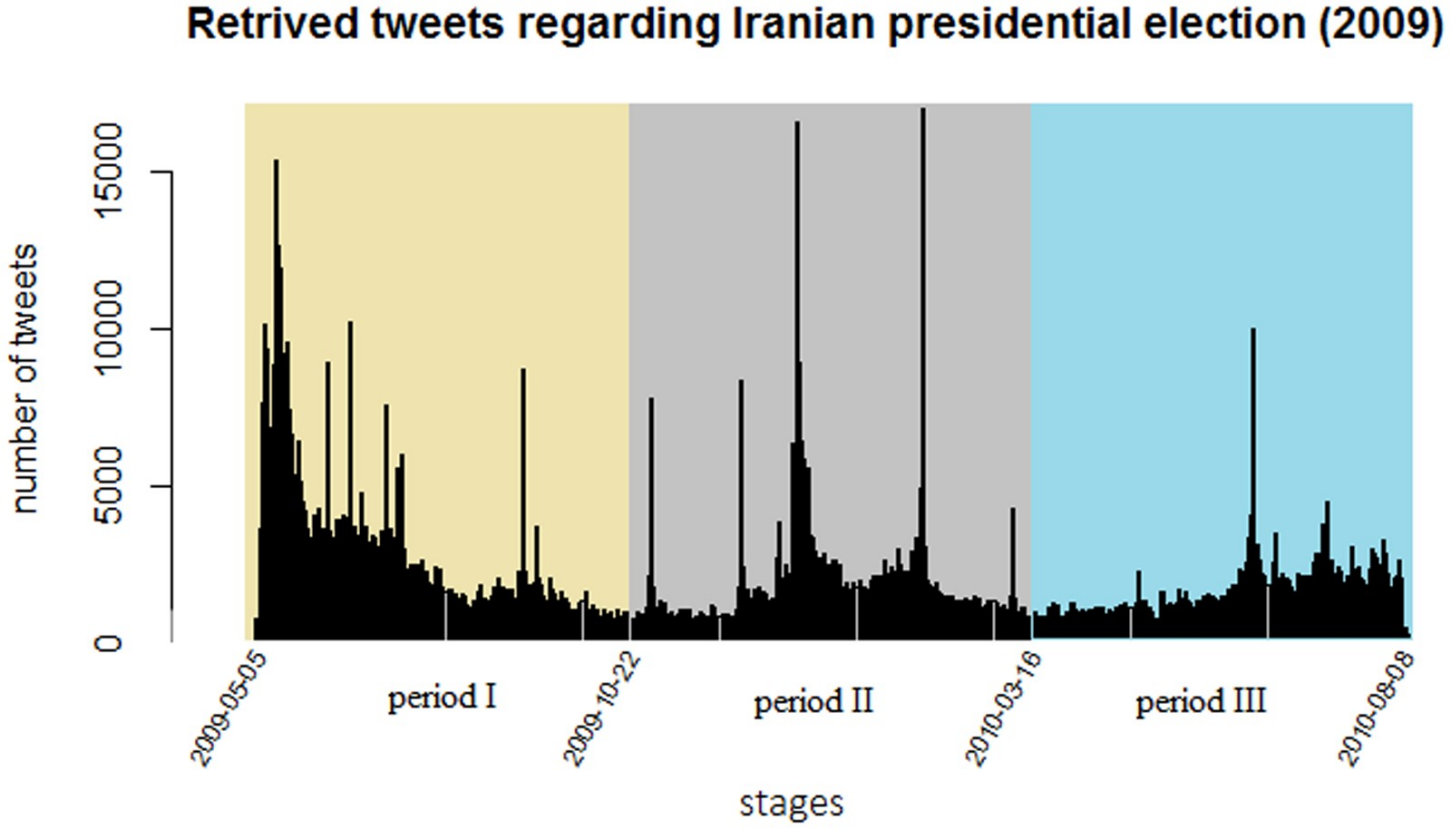
Tweets Frequency. Over time frequency of tweets regarding Iranian presidential election (2009). The whole period is divided into three equal length periods. High peaks majorly refer to important events of the time such as mass rallies and protests.

The data consist of tweets pertaining to this presidential election and the follow-up events that were previously collected and studied in [23]. This amounts to 1,532,289 tweets in total that were retrieved from one month before the elections until roughly 15 months after, to be precise: from 5 May 2009 until 8 August 2010. Duplicated and empty tweets, e.g. tweets containing only website links, were removed, resulting in 1,004,428 remaining tweets. We divided these 15 months into 3 periods of roughly 5 months each. Fig 11 presents the frequency of the tweets per day.

Next, the tweets in each period were divided into *n*_*t*_ = 500, *t* = 1, 2, 3 ‘bags of words’ or so called *documents*. These were subjected to standard text normalization techniques such as stemming and space/stopword/punctuation removal. We first identified the most frequent terms shared between the 3 periods and selected *p* = 30 co-occurring terms. Then, for each period a *term-document matrix* with rows and columns representing documents and (shared) terms, respectively, was created. Finally, the term-document matrices were mapped into continuous valued data matrix **Y** of the three periods *t* = 1, 2, 3 with, for *i* = *n*_<*t*_ + 1, …, *n*_<*t*_ + *n*_*t*_, and *t* = 1, 2, 3,

$$\begin{array}{ccc} Y_{i} & = & \{tf_{i}\left(w_{1}\right)\;\text{log}\left[n_{t}/df\left(w_{1}\right)\right],\ldots ,tf_{i}\left(w_{p}\right)\;\text{log}\left[n_{t}/df\left(w_{p}\right)\right]\}\;\;\text{for} \end{array}$$

where *tf*_*i*_(*w*_*j*_) represents the *term frequency* of *w*_*j*_, this is the frequency with which the word *w*_*j*_ occurs in the *i*-th document, and *df*(*w*_*j*_) the *document frequency* of *w*_*j*_, this is number of documents in which *w*_*j*_ appears.

We constructed the similarity matrix $S^{t}={\left(s_{ii^{\prime }}^{t}\right)}_{i\leq i^{\prime }=1}^{n_{t}}$ based on the number of days between the posting dates of tweets. The impact of this measure on the clustering in mixture models is studied in an earlier paper [24] where several similarity measures are compared.

#### 3.2.3 Results

The primary purpose of this analysis was to reconstruct networks that signify important topics in each period through linking terms or words. Different versions of the Bayesian fused graphical lasso estimation that were presented in the preceding sections, as well as the Bayesian lasso, were applied to the data. For clarity these are briefly recapped:

non-mixture: Data of each period are assumed to follow a multivariate normal distribution and their precision matrices are estimated by the Bayesian graphical lasso [7].
BSM-CRP: To account for heterogeneity, data from each period are assumed to follow the mixture model (12). The Bayesian stage-wise mixture (BSM) algorithm is used to estimate the model parameters. This algorithm uses a data allocation scheme that is equivalent to the CRP.
BSM-DICRP: Identical to the BSM-CRP approach but with DIRCP used for data allocation in which the number of days between documents serves as external information (see Section 2.2.1). The latter accommodates the possibility of documents contiguous in time to entail more similar information than documents well-separated in time.
BFM-CRP: As before a mixture model per period is assumed to be a good description of the data. In the estimation of these mixture models the precision matrices of the mixture components may now inherit or share structure within or between time periods. This is achieved by the Bayesian fused graphical (BFM) mixture approach (see Section 2.2.3). The CRP is used for data allocation in the mixture estimation.
BFM-DICRP: As BFM-CRP: but with the DICRP used for data allocation in which the number of days between days serves as external information.

**Fig 12:**
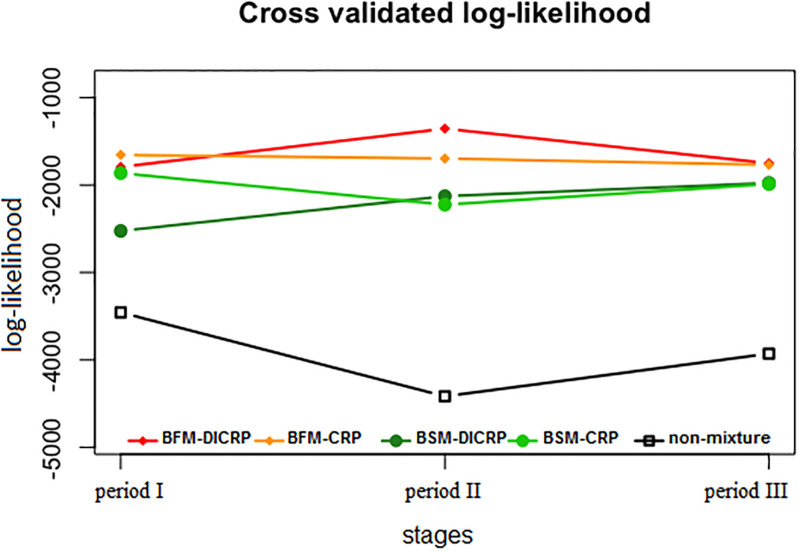
Performance of estimation approaches. Predictive log-likelihoods obtained by 5-fold cross-validation corresponding to three stage twitter data analysis with a non-mixture estimation, mixture estimations without additional similarity data (BSM-CRP and BFM-CRP), and mixture estimations taking into account external information on similarity of consequent tweets (BSM-DICRP and BFM-DICRP).

These methods are compared for the Twitter data and compared with respect to their predictive power established through a 5-fold cross-validation method. The hyperparameter values are equal to those described in the simulation section. The results of this comparison study are summarized in Fig 12.

**Fig 13:**
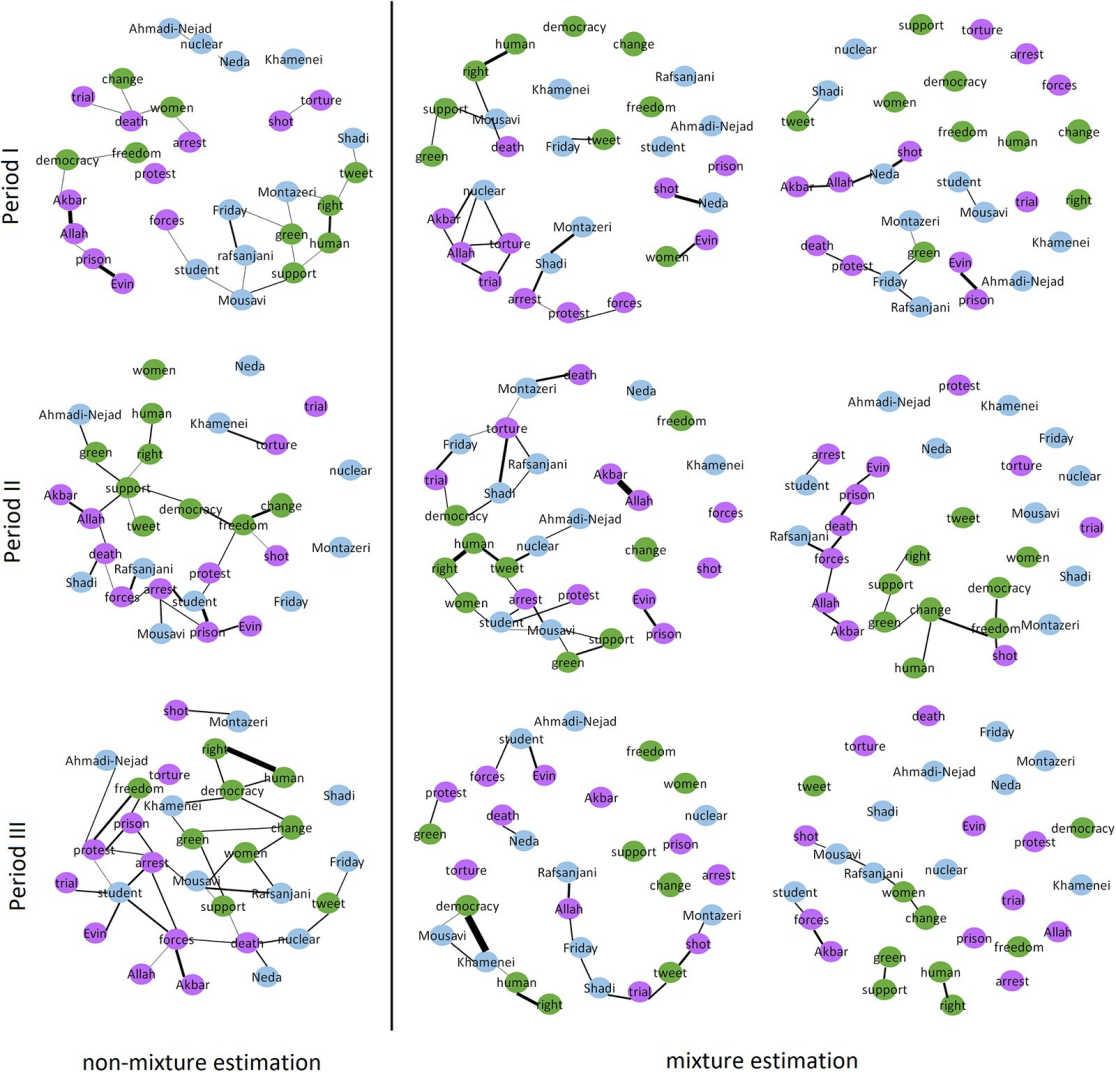
Reconstructed networks of terms. Reconstructed term networks from tweets regarding Iranian presidential elections 2009, using a BSM-DICRP and non-mixture estimation approaches.

A first observation inferable from Fig 12 is the substantial difference between non-mixture and mixture estimation methods. The heterogeneity assumption thus seems to be a crucial one. Secondly, among the mixture estimation approaches those equipped with a DICRP data allocation scheme reveal a slight estimation improvement compared to the approach with the CRP scheme. This suggests that information of the number of days between tweets aids in the mixture component assignment of individual terms. Finally, the BFM approaches show a slight performance improvement, in terms of prediction accuracy, over their BSM counterparts. Final results of the Twitter data are thus based on the BFM-DICRP analysis, which are contrasted to those originating from the non-mixture approach. The corresponding networks are displayed in Fig 13. These are based on the partial correlation matrices obtained by standardization from the estimated precision matrices. The networks comprise 30 terms (nodes) linked by edges whose 95% Bayesian credible interval does not contain zero.

The resulting networks of Fig 13 reveal groups of terms or words that are more frequently linked within than between groups:

1. (*Colored green*.) The key words of speeches, open statements of political leaders, or demands of protesters such as ‘human’, ‘right’, ‘change’, ‘support’, ‘green’ (for “Green Movement”), ‘democracy’, ‘tweet’, ‘women rights’, ‘freedom’.
2. (*Colored purple*.) The key words that can either represent strict measures taken by the government to suppress the protests such as ‘torture’, ‘prison’, ‘Evin’ (name of a prison in Tehran), ‘protest’, ‘shot’, ‘forces’, ‘arrest’, ‘trial’, or chanting words such as ‘Allah’, ‘Akbar’, ‘death’.
3. (*Colored blue*.) The names of significant political leaders or influential groups/individuals such as ‘Ahmadi-Nejad’, ‘Khamenei’, ‘Montazeri’, ‘Mousavi’, ‘Neda’, ‘Rafsanjani’, ‘Shadi’, ‘student’, and ‘women’.

This grouping is best recognized by mixture estimation, particularly for the first and second period. This may be due to the fact that most demonstrations and the subsequent reaction by the police took place in the first two time periods.

The reconstructed networks of words across tweets reflect the evolution of the Iranian political situation at the time. Several pairs of words, such as ‘human–right’, ‘Evin–prison”, ‘Allah–Akbar’, ‘support–green (movement)’ are connected in the networks of all three periods. Both words within each pair stem from the same semantic category, irrespective of the method used. Such a semantic grouping is, however, more prominent in the results of period I and II from the mixture model. This is most likely due to the fact that these two periods saw most demonstrations, with possible reaction of the state. More specifically, period-specific meaningful, i.e., coinciding with the events listed in the above presented time line, links can be identified from the reconstructed networks. For example, the ‘Neda’-‘shot’ and ‘Shadi’-‘arrest’ link appears only in the first time period. Further, the ‘Montazeri–death’ link in the period II networks reflect the death of Ayatollah Montazeri during this period. Finally, the word ‘student’ takes a more central place in the period II networks due to two student events, related arrests and claimed torture of students that took place then.

The mixture modeling identifies two different groups of tweets for each period (see Fig 13). These groups may be interpreted by means of the cohesion among words and their semantics. For example, in period I the conditional independence graph of the first mixture component reveals word clusters that broadly combine topics of “public tumult” and “political demands”, while that of the other mixture component appears to connect words only if they relate to actual events. As such the two groups may loosely represent tweets originating from twitter accounts aliased with the protest and media, respectively. For period II a different contrast between the cohesion of words within tweets becomes apparent. The first mixture component is hard to interpret and may represent a mixed bag of tweets representing the general turmoil of the period. The second mixture component, however, nicely shows two distinct clusters each representing a different semantic category: tweets with a single uniform message filled either with the protester’s demands or with an account of the negative events taking place.

## 4 Discussion

This paper transfers and extends the Bayesian graphical lasso for network reconstruction to the field of (chronological) textual social media data analysis. Twitter data from several time periods related to the 2009 Iranian presidential elections are used to show the potential of the approach. The data are studied from a graphical network estimation perspective and identifies the relation (and their variation over time) among topics. Statistically, the problem amounts to simultaneous estimation of precision matrices which is solved by the Bayesian graphical lasso, and which is extended here to *i)* account for heterogeneity in the data, *ii*) incorporate external information in the unravelling of this heterogeneity, and *iii)* borrow network similarities among identified groups. Extraction of summary information from one and a half million tweets related to the aforementioned election shows promise. Moreover, the flexibility of this Bayesian framework enables several approaches to address different assumptions on the data structure.

A possible useful inroad for future research might be to address high-dimensionality. The presented Twitter data analysis was limited to *p* = 30 terms. The proposed method is applicable to larger *p*, but an increase of the dimension may prohibit the interpretation of the reconstructed network. For comprehensive interpretation of large networks it might be crucial to develop a complementary semantic analysis, possibly based on a community finding method.

## Supporting information

S1 Data (ZIP)

S1 Table Parameter values used in the second simulation study. (PDF)
