# Supplementary material for: Analysis of Twitter data with the Bayesian fused graphical lasso
Source: PLoS One. 2020 Jul 27;15(7):e0235596. doi: 10.1371/journal.pone.0235596 (PMC7384635; doi:10.1371/journal.pone.0235596)
Supplement: S1 Table — (PDF) [file pone.0235596.s002.pdf]

Supporting Information

Simulation settings

Table 1 presents component-wise mean and mixing probability parameters of the mixture models for stages 2 and 3, and non-mixture model parameters corresponding to stage 1.

Stages	π	μ
I	1	$\boldsymbol{\mu}_{1,1} = (1, 1, 1, .., 1, 1)_{1\times p}$
II	(1/2,1/2)	$\boldsymbol{\mu}_{2,1} = (-3, -3, -3, .., -3, -3)_{1\times p}$
		$\boldsymbol{\mu}_{2,2} = (3, 3, 3, .., 3, 3)_{1\times p}$
III	(1/3,1/3,1/3)	$\boldsymbol{\mu}_{3,1} = (-3, -3, -3, .., -3, -3)_{1\times p}$
		$\boldsymbol{\mu}_{3,2} = (0, 0, 0, .., 0, 0)_{1\times p}$
		$\boldsymbol{\mu}_{3,3} = (3, 3, 3, .., 3, 3)_{1\times p}$

Table 1. Parameter values used in the second simulation study.

We considered the following parametrization for the partial correlation matrices from which covariance matrices were calculated. Fig 3 illustrates the corresponding covariance matrices.

1. $\mathbf{P}_{v_{1,1}}$: $\rho_{jj} = 0.7$, $\rho_{j_1 j_2} = \rho_{ji} = 0.4$ for $i, j \leq p/2$ or $i, j > p/2$, and $\rho_{j_1 j_2} = 0$ otherwise.

2. $\mathbf{P}_{v_{2,1}}$: $\rho_{jj} = 0.9$, $\rho_{i,i-1} = \rho_{i-1,i} = 0.6$, $\rho_{i,i-2} = \rho_{i-2,i} = 0.4$, $\rho_{i,i-3} = \rho_{i-3,i} = 0.2$, $\rho_{i,i-4} = \rho_{i-4,i} = 0.1$, and $\rho_{j_1 j_2} = 0$ otherwise.

3. $\mathbf{P}_{v_{2,2}}$: $\rho_{jj} = 0.7$, $\rho_{j_1 j_2} = \rho_{ji} = 0.4$ for $i, j > p/2$, and $\rho_{j_1 j_2} = 0$ otherwise.

4. $\mathbf{P}_{v_{3,1}}$: $\rho_{jj} = 0.9$, $\rho_{i,i-1} = \rho_{i-1,i} = 0.6$, $\rho_{i,i-2} = \rho_{i-2,i} = 0.4$, and $\rho_{j_1 j_2} = 0$ otherwise.

5. $\mathbf{P}_{v_{3,2}}$: A randomly generated sparse positive definite matrix

6. $\mathbf{P}_{v_{3,3}}$=$\mathbf{P}_{v_{2,1}}$.
